# Methylation status of *nc886* epiallele reflects periconceptional conditions and is associated with glucose metabolism through nc886 RNAs

**DOI:** 10.1186/s13148-021-01132-3

**Published:** 2021-07-22

**Authors:** Saara Marttila, Leena E. Viiri, Pashupati P. Mishra, Brigitte Kühnel, Pamela R. Matias-Garcia, Leo-Pekka Lyytikäinen, Tiina Ceder, Nina Mononen, Wolfgang Rathmann, Juliane Winkelmann, Annette Peters, Mika Kähönen, Nina Hutri-Kähönen, Markus Juonala, Katriina Aalto-Setälä, Olli Raitakari, Terho Lehtimäki, Melanie Waldenberger, Emma Raitoharju

**Affiliations:** 1 Department of Clinical Chemistry, Finnish Cardiovascular Research Center Tampere, Faculty of Medicine and Health Technology, Tampere University, Pirkanmaa Hospital District and Fimlab Laboratories, Tampere, Finland; 2 Gerontology Research Center, Tampere University, Tampere, Finland; 3 Heart Group, Finnish Cardiovascular Research Center, Tampere, Faculty of Medicine and Health Technology, Tampere University, Tampere, Finland; 4 Research Unit Molecular Epidemiology, Helmholtz Zentrum München, German Research Center for Environmental Health, 85764 Neuherberg, Bavaria Germany; 5 Institute of Epidemiology, Helmholtz Zentrum München, German Research Center for Environmental Health, Neuherberg, Bavaria Germany; 6 German Center for Diabetes Research (DZD), Munich, Neuherberg, Germany; 7 Institute for Biometrics and Epidemiology, German Diabetes Center, Leibniz Center for Diabetes Research At Heinrich Heine University, Düsseldorf, Germany; 8 Medical Faculty, Heinrich Heine University, Düsseldorf, Germany; 9 Institute of Neurogenomics, Helmholtz Zentrum München, German Research Center for Environmental Health, Neuherberg, Germany; 10 Department of Neurogenetics and Institute of Human Genetics, Technical University of Munich, Munich, Germany; 11 German Centre for Cardiovascular Research (DZHK), Partner Site Munich Heart Alliance, Munich, Germany; 12 Department of Clinical Physiology, Faculty of Medicine and Health Technology, Tampere University and Tampere University Hospital, Tampere, Finland; 13 Tampere Centre for Skills Training and Simulation, Tampere University, Tampere, Finland; 14 Division of Medicine, Department of Medicine, Turku University Hospital, University of Turku, Turku, Finland; 15 Centre for Population Health Research, University of Turku, Turku University Hospital, Turku, Finland; 16 Research Centre of Applied and Preventive Cardiovascular Medicine, University of Turku, Turku, Finland; 17 Department of Clinical Physiology and Nuclear Medicine, University of Turku, Turku University Hospital, Turku, Finland; 18 Heart Hospital, Tampere University Hospital, Tampere University, Tampere, Finland

**Keywords:** nc886, vtRNA2-1, miR-886, Genomic imprinting, Population studies

## Abstract

**Background:**

Non-coding RNA 886 (*nc886*) is coded from a maternally inherited metastable epiallele. We set out to investigate the determinants and dynamics of the methylation pattern at the *nc886* epiallele and how this methylation status associates with nc886 RNA expression. Furthermore, we investigated the associations between the *nc886* methylation status or the levels of nc886 RNAs and metabolic traits in the YFS and KORA cohorts. The association between *nc886* epiallele methylation and RNA expression was also validated in induced pluripotent stem cell (iPSC) lines.

**Results:**

We confirm that the methylation status of the *nc886* epiallele is mostly binomial, with individuals displaying either a non- or hemi-methylated status, but we also describe intermediately and close to fully methylated individuals. We show that an individual’s methylation status is associated with the mother’s age and socioeconomic status, but not with the individual’s own genetics. Once established, the methylation status of the *nc886* epiallele remains stable for at least 25 years. This methylation status is strongly associated with the levels of nc886 non-coding RNAs in serum, blood, and iPSC lines. In addition, *nc886* methylation status associates with glucose and insulin levels during adolescence but not with the indicators of glucose metabolism or the incidence of type 2 diabetes in adulthood. However, the nc886-3p RNA levels also associate with glucose metabolism in adulthood.

**Conclusions:**

These results indicate that *nc886* metastable epiallele methylation is tuned by the periconceptional conditions and it associates with glucose metabolism through the expression of the ncRNAs coded in the epiallele region.

**Supplementary Information:**

The online version contains supplementary material available at 10.1186/s13148-021-01132-3.

## Background

Non-coding RNA 886 (*nc886,* VTRNA2-1 as its HGNC symbol) is encoded in chromosome 5q31.1, from a 1.9-kb long, differentially methylated region (DMR), the boundaries of which are marked by two CCCTC-binding factor (CTCF) binding sites [1–3]. This DMR has been reported to function as a metastable epiallele [4, 5], with maternal imprinting in 75% of individuals in several populations [1]. This means that, while in all individuals the paternal allele is non-methylated, in 75% of individuals the maternal allele is methylated, and the individuals are hemi-methylated in this locus. In the remaining 25% of individuals, also the maternal allele is non-methylated, and these individuals are non-methylated in this locus. The methylation status of the *nc886* locus has been shown to be stable for at least 10 years, and it is similar in all tested tissues of one individual [4]. It has been indicated that the methylation of the maternal *nc886* epiallele is a post-fertilization event, occurring early in development [6].

The nature of the RNA products transcribed from the *nc886* gene remains ambiguous [7–9]. Initially, the non-coding RNA was known as pre-miR-RNA 886, producing miR-886-5p and miR-886-3p [10]. Later, the RNA was suggested to be a vault RNA (vtRNA2-1) or short vault RNA (svtRNA) [9–11], but subsequent evidence again supports the existence of a full-length nc886 (101/102 nt) [12] and two short RNAs produced from it (hsa-miR-886-3p/nc886-3p [23 nt] and hsa-miR-886-5p/nc886-5p [24–25] nt]) [8, 13, 14]. In this study, these RNAs are referred to as nc886-3p, nc886-5p, and nc886-102 nt, and the term short nc886 RNAs is reserved for nc886-3p and -5p alone.

The nc886-102 nt is highly abundant and expressed in almost all cell types [15]. It has a short half-life of only about an hour and is localized in the cytoplasm in a regulated manner [6]. The functions of the nc886 RNAs are under investigation. nc886-102nt has been shown to inhibit the protein kinase R (PKR) and has been suggested to take part in tumor surveillance [16]. Furthermore, both nc886-3p and -5p have been linked to the progression of cancer [8, 17]. The expression of nc886-102 nt has been shown to be regulated by the methylation status of the *nc886* epiallele in blood cells [18], and the removal of DNA methylation has been shown to increase nc886-3p expression in cancer cell lines [19]. However, studies on large population-based samples and non-cancerous cell lines examining the association of the *nc886* epiallele methylation status with the expression levels of different *nc886-*locus-derived RNAs are still missing. As the *nc886* gene is present only in primates and guinea pigs [1], there is also no data from model organisms exploring the relationship between *nc886* methylation and RNA expression.

The periconceptional environment has been suggested to affect DNA methylation patterns in maternal alleles [20], including the *nc886* epiallele [4]. For example, the season of conception [4], maternal levels of vitamin B2 [16] or methionine [4], mothers alcohol consumption [21], and the mother’s age [1, 21, 22] have been associated with the child’s *nc886* methylation status [4]. On the other hand, lower levels of *nc886* methylation have been linked to cleft palate [23], and a non-methylated *nc886* epiallele has been associated with an elevated childhood BMI [24]. The methylation status of this epiallele has also been associated with allergies [25], asthma [26], infections [27], and inflammation [28]. Previous studies have not found indications that genetics impact the methylation status of the *nc886* epiallele [4, 18, 21, 29], but a study has suggested that one SNP (rs2346018) is associated with the DNA methylation levels in the centromeric CTFC binding site flanking the *nc866* epiallele [1].

In mammalian genomic imprinting, only one parental allele is expressed, while gene expression from the other allele is suppressed in a parent-of-origin-dependent manner. The expression of imprinted genes in general has been associated with fetal and placental growth and suggested to a have a role in the development of cardiometabolic diseases in adulthood [30, 31]. Instead of classically imprinted genes, where the regulation is determined by the parental origin of the allele, metastable epialleles are defined as an “epiallele at which epigenetic state can switch and establishment is a probabilistic event” and “once established the state is mitotically inherited” [32]. As a metastable epiallele, *nc886* could mediate the association between periconceptional conditions and later metabolic health, in line with the Developmental Origins of Health and Disease (DOHaD) hypothesis (aka the Barker hypothesis) [33].

This research was set up to investigate if and how the DMR overlapping *nc886* acts as a tunable metastable epiallele, and whether the *nc886* epiallele methylation status is associated with metabolic traits after infancy. In more detail, we characterized the methylation status of the *nc886* epiallele and investigated the long-term dynamics of this pattern in population-based follow-up cohorts of the Young Finns study (YFS) and the Cooperative Health Research in the Region Augsburg (KORA) cohort. We further describe if and how individuals’ genetics and periconceptional conditions associate with *nc886* epiallele methylation status and the manner in which the methylation status associates with the genome wide gene expression and nc886 RNA expression in the YFS. We also set out to verify the association between *nc886* epiallele methylation status and nc886 RNA expression in an induced pluripotent stem cell (iPSC) model. Finally, we investigated whether *nc886* methylation status or the levels of nc886 RNAs associate with metabolic traits in population-based study cohorts.

## Results and discussion

### The *nc886* epiallele presents a stable categorical methylation pattern

**Fig. 1:**
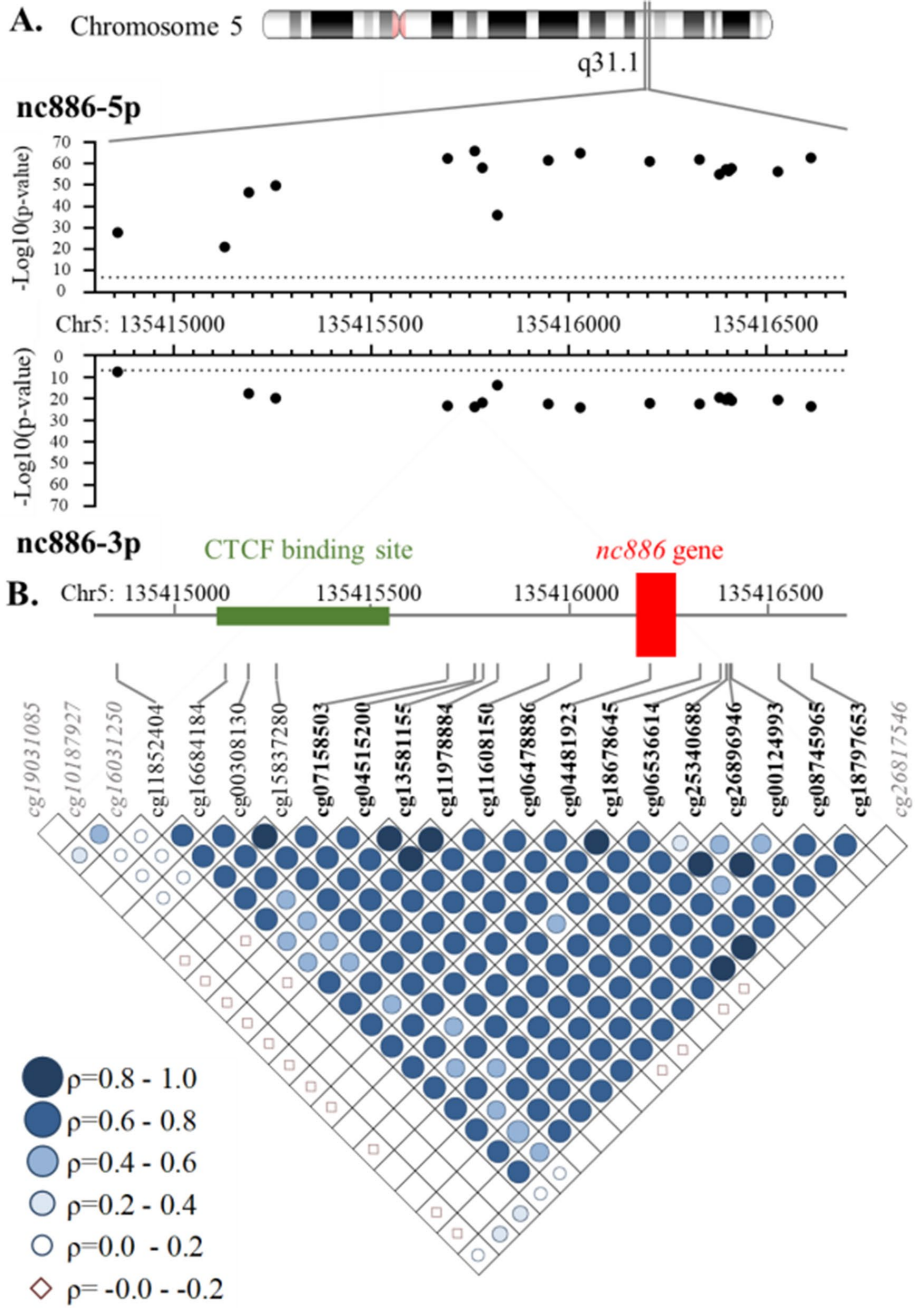
CpG sites associated with whole blood RNA levels of nc886-3p and nc886-5p. Methylation and sncRNA data were available for 806 and 825 individuals, respectively. **a** Chromosomal location and statistical significance of CpG sites within the 1.9 kb *nc886* DMR. **b** Correlations between all CpG sites that were associated with either nc886-3p or nc886-5p RNA levels. CpG sites outside the DMR are shown in grey italics and CpGs within the DMR in black. In boldface are the CpG sites that display a bimodal methylation pattern, based on which the individuals were categorized as non-, intermediately, or hemi-methylated. The methylation *β* values for the 14 bimodal CpG sites are presented in Additional file 1: Figure S2

Our aim was to verify the existence of the *nc886* DMR and to define the *nc886* epiallele in a population-based YFS cohort (Additional file 1: Figure S1). To this end, we performed an epigenome-wide association study (EWAS; *n* ~ 800) in the YFS to identify all CpGs in which the level of methylation is associated with the level of either nc886-3p or nc886-5p in whole blood. We identified 21 CpGs associated with nc886-5p and 19 CpG sites associated (*p* < 5 × 10^–8^) with nc886-3p blood RNA levels (Additional file 1: Table S1)—17 and 18 of these CpG sites, respectively, were located within the previously described 1.9 kb DMR [1] (Fig. 1). The CpGs outside the DMR were located in the *IL9* gene (nc886-3p and nc886-5p) or in and upstream of the *TGFBI* gene (nc886-3p). The methylation levels of the CpG sites located within the *nc886* DMR correlated well with each other, but not with the CpG sites outside the DMR (Fig. 1).

**Fig. 2:**
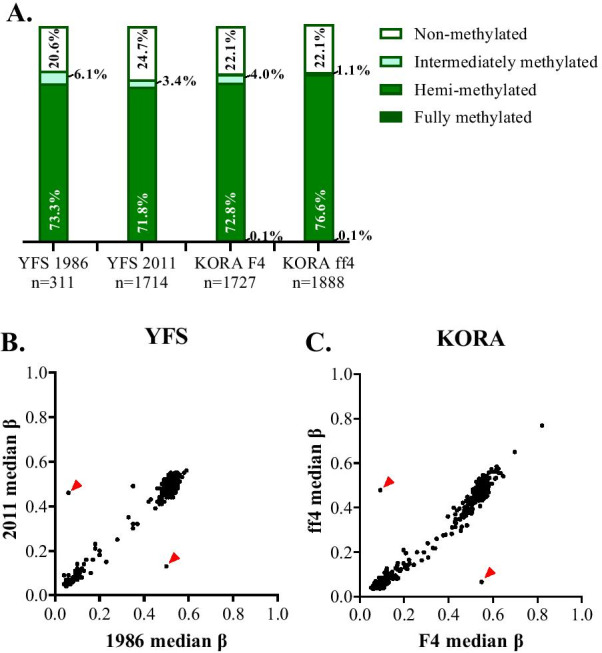
Methylation status of the *nc886* epiallele in population cohorts. **a** Distribution of the *nc886* epiallele methylation status groups in two cohorts, the YFS and the KORA, at two time points (KORA F4/2007, KORA FF4/2014). **b** and **c** Stability of the *nc886* epiallele methylation level (median of the 14 bimodal CpG sites) across two time points in the YFS (overlap between time points *n* = 309) and the KORA (overlap between time points *n* = 988). In both cohorts, we identified two individuals whose methylation levels seem to have changed between time points (indicated with red arrows). These individuals were excluded from further analyses, see Additional file 1: Methods

In line with the literature published previously [1, 34], 14 of the CpGs in the *nc886* DMR presented a bimodal distribution in our population (Additional file 1: Figure S2A). These CpGs were further used to divide the individuals from the YFS and the KORA cohorts into three groups with hierarchical clustering. The individuals were classified as non-methylated (with the median methylation level [*β* value] of the selected 14 CpGs ~ 0.10), hemi-methylated (*β* value ~ 0.50), and intermediately methylated (~ 0.35) (Additional file 1: Figure S2B and C). Although most of the individuals with intermediately methylated *nc886* DMR present methylation β levels between 0.25 and 0.45, this group is distinguished more by the particular methylation profile and the variance between probes than specific methylation level. The terms *non-methylated*, *intermediately methylated,* and *hemi-methylated* are hereafter used to refer to these groups. The prevalence of these groups corresponded to those reported previously [1] (Fig. 2a).

Silver et al. have described that the observed methylation status remains stable during a 10-year follow-up, from age 7–17 [4]. To verify this, we utilized methylation data from the YFS (two time points, 1986 [*n* = 311] and 2011 [*n* = 1714], with an overlap of 309 individuals) and from the KORA (two time points; F4/2007 [*n* = 1727] and FF4/2014 [*n* = 1888], with an overlap of 988 individuals). The median methylation level of the selected 14 CpGs was strongly correlated between the two time points in both cohorts (*p* = 7.18 × 10^–73^, *ρ* = 0.802, and *p* = 8.13 × 10^–206^, *ρ* = 0.783, respectively) (Fig. 2b), and the methylation status of the *nc886* epiallele is well preserved during the follow-ups (Fig. 2b, c). We thus show that the methylation status of the *nc886* epiallele also remains stable in adulthood. As metastable epialleles are defined as epigenetic states whose establishment is a probabilistic event and whose status is mitotically stable [32], these results show that the *nc886* DMR functions as a metastable epiallele.

In addition to the non- and hemi-methylated groups, we describe a group of individuals (1–6% of the population) presenting an intermediately methylated *nc886* epiallele. This methylation status is also well preserved through the 25 years of follow-up, and the finding is in line with the report by Carpenter et al. [1], who classified these kinds of samples as “inconclusive.” As the existence of the intermediately methylated group is not straightforwardly explained by the proposed methylation pattern at the *nc886* epiallele, we wanted to replicate the methylation analysis with a separate method. The methylation level of an approximately 250-nt area in the *nc886* epiallele was successfully profiled with a mass spectrometry–based bisulfite sequencing method (EpiTyper) from 12 individuals of the YFS cohort, presenting a stable methylation status from 1986 to 2011. These results replicate the median methylation levels of the profiled individuals and thus showed that the existence of the intermediately methylated individuals did not depend on the DNA methylation profiling method (Additional file 1: Figure S3). Our data therefore suggest that the intermediate methylation status is a real biological phenomenon and not a technical artifact.

One CpG site can present only 0% (no), 50% (monoallelic), or 100% (biallelic) methylation in one cell. Therefore, individuals with the intermediately methylated *nc886* epiallele should theoretically harbor a mixed population of hemi- and non-methylated cells. This could arise due to different cell lineages presenting different methylation statuses. To further investigate this possibility, we analyzed the data of Rhead et al. [35] comprising DNA methylation data from monocytes, B cells, and naïve and memory T cells from the same individuals. In these data, the median methylation levels at the *nc886* epiallele correlated strongly between the cell types (*ρ* > 0.6), and individuals presenting the intermediate methylation pattern in one cell type also present this methylation pattern in other cell types (Additional file 1: Figure S4). This intermediate methylation pattern could arise from stochastic methylation of the epiallele in different individual cells during early embryonic development, but the determinates leading to this in some individuals is outside the reach of this study. Our findings are also supported by the report by Treppendahl et al. [18], showing that different blood cell lineages present a similar *nc886* epiallele methylation status. Furthermore, Silver et al. [4] have reported that tissues arising from different germ layers present similar *nc886* epiallele methylation statuses, supporting the idea that the methylation pattern of the *nc886* epiallele is independent of cell type.

We also identified another exception to the binomial methylation pattern, as in the KORA cohort, we detected two individuals with a methylation level of above 60% in the *nc886* epiallele in both follow-ups (Fig. 2a, c). This implies that the paternal allele has also been methylated, at least in some proportion of the cells, which has not previously been described in the *nc886* epiallele in peripheral blood samples. It remains to be investigated when and how these newly described *nc886* methylation patterns arise and what their associations with an individual’s health are.

### Circulatory nc886 RNA levels are associated with *nc886* epiallele methylation status in the YFS

**Fig. 3:**
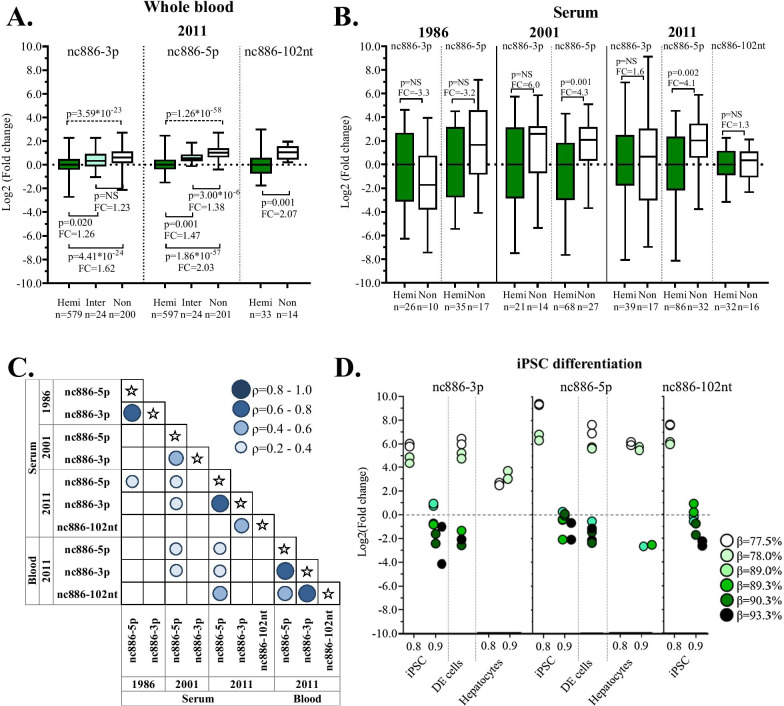
Association between levels of nc886 RNAs and the epiallele methylation. RNA levels of nc886-3p, nc886-5p, and nc886-102nt are higher in individuals with a non-methylated (Non) than with a hemi-methylated (Hemi) *nc886* epiallele in **a** whole blood and **b** in serum of the YFS cohort. Notably, those with an intermediately methylated *nc886* epiallele (Inter in **a**) also have intermediate nc886 RNA levels. **c** Between tissue and time point correlation were mostly seen with nc886-5p RNA levels. **d** In iPSCs, we detected two expression clusters in all nc886 RNAs. Also, in iPSCs, the expression level was associated with *nc886* epiallele methylation status (visualized as the color gradient, with color intensity increasing with increasing methylation level, the methylation beta values for iPSC and hepatocytes are also indicated on the *X*-axis). Furthermore, we see a trend towards decreasing nc886 RNA expression as the cells are differentiated from iPSCs into definitive endoderm (DE) cells and hepatocytes. Between-group comparisons analyzed with the Mann–Whitney test are presented by solid lines and those with the Kruskal–Wallis test over different methylation status groups by dotted lines in (**a**) and (**b**)

The *nc886* epiallele methylation status can be considered a categorical variable (non-, intermediately, and hemi-methylated), and, therefore, we re-analyzed the association between the expression of nc886 RNAs in the blood and *nc886* epiallele methylation status accordingly. In whole blood in the YFS, the expression levels of nc886-3p and nc886-5p were higher in individuals with a non-methylated epiallele, when compared to those with a hemi-methylated epiallele (*p* = 4.41 × 10^–24^, fold change [FC] = 1.62, *n* = 779, and *p* = 1.86 × 10^–57^, FC = 2.03, *n* = 797, respectively). Interestingly, in the intermediately methylated group, the blood RNA levels of nc886-3p and -5p were higher than in the hemi-methylated group but lower than in the non-methylated group, further validating the existence of a small group of individuals escaping the dichotomous methylation pattern (Fig. 3a). The level of nc886-102nt was also significantly higher in the blood of individuals with a non-methylated *nc886* epiallele compared to those with a hemi-methylated epiallele (*p* = 0.001, FC = 2.07, *n* = 47) (Fig. 3a). Treppendahl et al. have previously described similar expression pattern of nc886-102nt (vtRNA2-1) in the peripheral monocytes of 20 individuals [18].

In the YFS, serum short nc886 RNA expression data were available from three time points (1986, 2001, and 2011). At all time points during the 25-year follow-up, the nc886-5p RNA levels were higher in non-methylated than in hemi-methylated individuals, with fold changes ranging from 3.2 to 4.3 (*p* < 0.05) (Fig. 3b). A similar but statistically nonsignificant pattern can be seen for nc886-3p. The median RNA levels of nc886-102nt were also up-regulated in individuals with a non-methylated epiallele when compared to those with a hemi-methylated epiallele in the 2011 samples, but the difference was not statistically significant (Fig. 3b).

The levels of the three different nc886 RNAs correlate with each other in whole blood (Fig. 3c, Additional file 1: Figure S5A and Table S2A). In serum, nc886-3p and nc886-5p were correlated at all three time points (*p* < 0.05), but nc886-102nt (data only from 2011) correlated with only nc886-3p (Fig. 3c, Additional file 1: Figure S5B and Table S2A). In line with the results showing stable methylation levels of the *nc886* epiallele during the 25 years of follow-up, the RNA levels of nc886-5p showed good correlation from year to year as well as between serum and blood. However, for nc886-3p, we identified no correlation between the years or between blood and serum.

The literature is inconclusive as regards the nature of the nc886 RNAs [7–9]. All three nc886 RNAs have been reported to be functional molecules [8, 9], with discrepancies as to whether or not the short forms, nc886-3p and nc886-5p, function as or akin to miRNAs [18], with the most recent paper coming to the conclusion that “…nc886(-102nt) is a precursor of two small RNAs that meet key microRNA criteria…” [8]. The levels of all three RNA species show correlation in our data, but not to a point that would suggest that all or some of the assays would measure the same molecule. We report our findings with the assumption that all three molecules exist and that all three are potentially functional and treat nc886-3p and -5p as functional miRNAs.

### Expression of nc886 RNAs is associated with *nc886* epiallele methylation level in iPSCs and iPSC-derived hepatocytes

The *nc886* epiallele methylation and nc886 RNA expression were further analyzed in six iPSC lines and in definitive endoderm (DE) cells, and hepatocytes derived from these lines. Three cell lines originated from individuals with T2D, while three were obtained from healthy controls. The methylation pattern profiled from iPSCs (day 0, d0) and hepatocytes (d19) did not follow the expected division into non- and hemi-methylated cell lines. All cell lines exhibited methylation levels of > 70% at the *nc886* epiallele. During differentiation into hepatocytes, the median methylation level at the *nc886* epiallele increased in 5 of the 6 cell lines studied (Additional file 1: Table S3). The discovered methylation pattern is well in line with the recent report by Yagi et al. [36], describing de novo DNA methylation at imprinted loci during reprogramming into naive and primed pluripotency. In more detail, Yagi et al. showed that maternally methylated DMRs, which present 50% methylation in somatic cells, have methylation levels of over 80% in iPSCs. Interestingly, data from Olsen et al. indicate that de novo methylation of the *nc886* epiallele could also occur in specific somatic cells in humans during aging [37] and in cancer cells, the methylation status frequently escapes imprinting [6, 12].

At the pluripotent stage (d0), we see strong correlation between all three RNAs (nc886-3p, nc886-5p, and nc886-102nt [measured only from d0 samples]) (Additional file 1: Table S2B). Binomial expression patterns can be seen throughout the differentiation process, even though nc886-3p and -5p RNA levels in DE and liver cells (d19) are too low to be detected in some of the cell lines (Fig. 3d). The association between methylation levels and RNA expression was also observed during iPSC differentiation into hepatocytes, when *nc886* epiallele methylation increased and the levels of nc886 RNA decreased (Fig. 3d). We thus confirm the association between the epiallele methylation status and RNA levels for the first time in a non-cancerous cell model with no extreme demethylating reagents (i.e., 5-azacytidine treatment).

### Pathway analysis of nc886-3p and nc886-5p target mRNAs reveals associations with endocytosis, insulin signaling, inositol phosphate metabolism, and myeloid leukemia pathways

To investigate the association of *nc886* methylation status and gene expression, we utilized the genome-wide gene expression profiling performed from the blood samples of the YFS 2011 follow-up (*n* = 1664). The methylation status of the *nc886* epiallele (non- vs. hemi-methylated) was not associated with the expression of any individual protein-coding gene or biological pathway at the level of FDR < 0.05. Genes and pathways associated with the *nc886* epiallele status at the level of nominal *p* < 0.05 are listed in Additional file 2: Tables S4 and S5. As we did not detect significant associations between *nc886* epiallele methylation status and the expression of protein-coding genes or pathways, our results suggest that the function of this metastable epiallele is specifically to regulate the expression of nc886 RNAs.

For the analyses between short nc886 RNA levels and gene expression, we utilized the target prediction of microRNA.org to identify the predicted mRNA targets of nc886-3p and nc886-5p RNAs. The RNA levels of nc886-3p in whole blood presented only nominally significant correlations with its predicted targets (Additional file 2: Table S6), while the RNA levels of nc886-5p in whole blood correlated with 95 targets at the level of FDR < 0.05 (Additional file 2: Table S7). Pathways in which the correlating target genes of either short nc886 RNAs were enriched are described in Table 1. Several of these pathways, including the *insulin-signaling pathway*, have been reported to be associated with nc886-3p also previously [8, 38]. In addition, the detected association between nc886-5p and the *acute myeloid leukemia pathway* is further supported by previous findings connecting *nc886* epiallele methylation and nc886-102nt expression to this disease [18]. These results give molecular insight into the possible functions of short nc886 RNAs. Furthermore, they link nc886 RNA expression in healthy individuals with cancer pathways, in the context of which *nc886* methylation and RNA expression have been widely studied [3, 7, 13–15, 18, 34, 38–42].

**Table 1:** KEGG and BIOCARTA pathways associated with nc886-3p and -5p expression

Gene set name	# Genes in gene set (K)	# Genes in Overlap (k)	*k*/K	*p* value	FDR *q* value
*nc886-3p*					
KEGG_ENDOCYTOSIS	181	8	0.044	4.37 × 10^–6^	0.002
KEGG_FC_GAMMA_R_MEDIATED_PHAGOCYTOSIS	96	6	0.063	9.99 × 10^–6^	0.002
KEGG_CHRONIC_MYELOID_LEUKEMIA	73	5	0.069	3.62 × 10^–5^	0.004
KEGG_INSULIN_SIGNALING_PATHWAY	137	6	0.044	7.46 × 10^–5^	0.005
KEGG_FOCAL_ADHESION	199	7	0.035	7.51 × 10^–5^	0.005
*nc886-5p*					
KEGG_INOSITOL_PHOSPHATE_METABOLISM	54	6	0.111	3.53*10^–7^	1.68 × 10^–4^
KEGG_PHOSPHATIDYLINOSITOL_SIGNALING_SYSTEM	76	6	0.079	2.72 × 10^–6^	0.001
KEGG_ACUTE_MYELOID_LEUKEMIA	57	5	0.088	1.13 × 10^–5^	0.002
*Both nc886-3p and nc886-5p*					
KEGG_ACUTE_MYELOID_LEUKEMIA	57	7	0.123	1.23 × 10^–6^	0.001
BIOCARTA_ERK_PATHWAY	27	5	0.185	5.36 × 10^–6^	0.001
KEGG_INOSITOL_PHOSPHATE_METABOLISM	54	6	0.111	1.31 × 10^–5^	0.002
KEGG_FC_GAMMA_R_MEDIATED_PHAGOCYTOSIS	96	7	0.073	4.04 × 10^–5^	0.004
KEGG_INSULIN_SIGNALING_PATHWAY	137	8	0.058	5.66 × 10^–5^	0.004
KEGG_PATHWAYS_IN_CANCER	325	12	0.037	8.20 × 10^–5^	0.004
KEGG_PHOSPHATIDYLINOSITOL_SIGNALING_SYSTEM	76	6	0.079	9.28 × 10^–5^	0.004
KEGG_FOCAL_ADHESION	199	9	0.045	1.42 × 10^–4^	0.006
KEGG_HEMATOPOIETIC_CELL_LINEAGE	87	6	0.069	1.96 × 10^–4^	0.008
KEGG_ENDOCYTOSIS	181	8	0.044	3.85 × 10^–4^	0.012
KEGG_CHRONIC_MYELOID_LEUKEMIA	73	5	0.069	0.001	0.021
BIOCARTA_MAPK_PATHWAY	81	5	0.062	0.001	0.028
KEGG_REGULATION_OF_ACTIN_CYTOSKELETON	213	8	0.038	0.001	0.028
KEGG_CHEMOKINE_SIGNALING_PATHWAY	189	7	0.037	0.002	0.044

Gene set enrichment was analyzed for genes that were predicted targets of nc886-3p/nc886-5p according to mircoRNA.org and whose RNA levels correlated (spearman rank order correlation) with the targeting ncRNA at the level of *p* < 0.05. Pathways with FDR < 0.05 and more than five genes overlapping are presented in the table

### Genetics regulate nc886 RNA expression and the methylation levels of individual CpGs in nc886 DMR but not the methylation status of the *nc886* epiallele

Previous studies [1, 21, 22] did not show associations of genetic polymorphisms with nc886 epiallele methylation status. Similarly, the GWAS, performed with *nc886* epiallele methylation status defined as non- versus hemi-methylated, in the YFS 2011 EPIC samples with genome-wide genotyping data available (*n* = 1264) did not present any significant (*p* < 5 × 10^–8^) associations between the methylation status and SNPs with minor allele frequency over 1%.

To further examine the effect of genetics on the DNA methylation of the nc886 DMR, we investigated the genetic association study results on the 18 individual CpGs located in the nc886 DMR in the Genetics of DNA Methylation Consortium (GoDMC) data (*n* > 32,000) and in YFS data (*n* = 1313). The four CpGs in the *nc886* DMR with a non-bimodal methylation pattern (cg11852404, cg16684184, cg00308130, and cg15837280) associated with a multitude of overlapping genetic variations in GoDMC cohorts (Additional file 1: Figure S6A and B). In YFS, only the methylation levels of cg16684184 and cg00308130 were associated with genetic variation, and the majority of these results were replicated in the GoDMC data (Additional file 1: Figure S6D, Additional file 2: Table S8). In both GoDMC and YFS data, rs2346018 was associated with the methylation levels of CpGs near the CTCF, representing a non-bimodal methylation pattern, as previously indicated by Carpenter et al. [1].

A considerably lower number of genetic variations were associated with the CpGs presenting a bimodal methylation pattern, and only 5 correlating SNPs associated with at least 4 of the 14 bimodally distributed CpGs in the GODMC data (Additional file 1: Figure S6A and C). In the YFS, we detected associations only between cg11978884 and 3 genetic variations, none of which are replicated in a larger data set (Additional file 1: Figure S6D, Additional file 2: Table S8). 6 CpGs overlapping or directly upstream of the nc886 gene did not associate with any genetic variation in either cohort, further indicating that *nc886* epiallele methylation status is not directly affected by genetics.

**Fig. 4:**
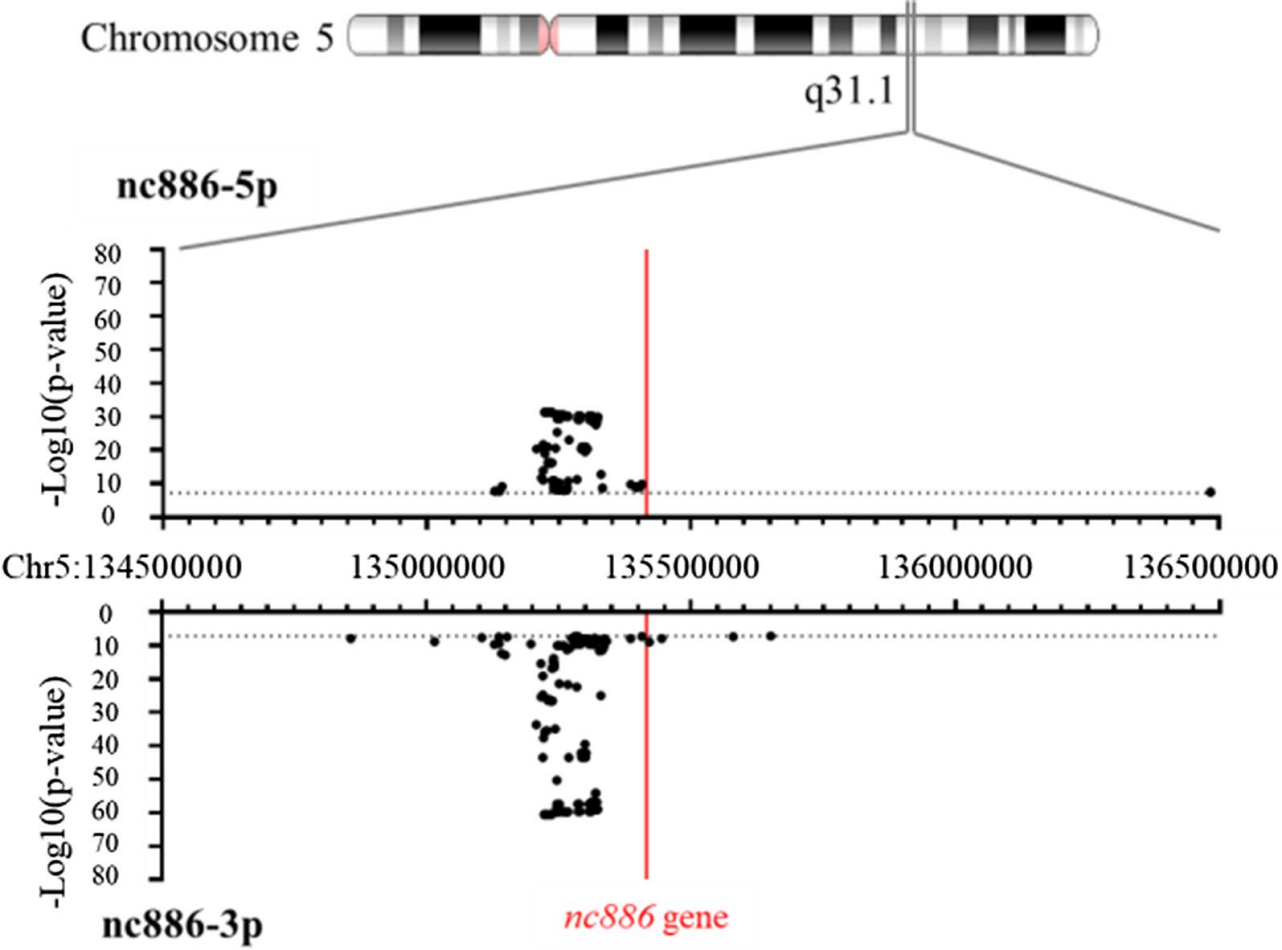
Results of the genome-wide association study on the nc886-3p and -5p RNA levels (*n* = 765). The majority of the SNPs associated with the short nc886 expression are located 100–200 kb down-stream of the *nc886* gene (*nc886* is transcribed in the reverse strand). In addition to the SNPs illustrated here, the nc886-3p RNA levels were associated with rs1027970117, a deletion in chromosome 15

We also performed a GWAS on the blood RNA levels of nc886-3p and -5p. Whole blood levels of nc886-3p and nc886-5p are associated (*p* < 5 × 10^–8^) with 180 and 130 overlapping genetic variations, respectively. Of these, all but one were located at chromosome 5. The genetic variations with the strongest association with nc886-3p and nc886-5p RNA levels were located within a 100-kb region from 92 to 193 kb downstream of the *nc886* gene (Fig. 4, Additional file 2: Tables S9 and S10). Interestingly, one of the CpG sites (cg19031085) outside the imprinted region, the methylation of which was associated with nc886-3p and -5p expression, is also located in this area, suggesting that this genetic region is important in the regulation of nc886 RNA expression.

Our results thus indicate that, even though genetics do not seem to have an effect on the establishment of *nc886* epiallele methylation status, genetics modulate the effects of the *nc886* epiallele through the regulation of nc886 RNA levels. Methylated *nc886* allele is not permissive for transcription, and this non-expressed status is unlikely to be affected by genetic variation. However, for the non-methylated *nc886* allele, that is permissive for transcription, the level of nc886 RNAs expression is further affected by a group of SNPs in the 100–200 kb region downstream of the *nc886 *loci.

### Maternal age and family socioeconomic status (SES) is associated with the methylation status of the *nc886* epiallele

**Fig. 5:**
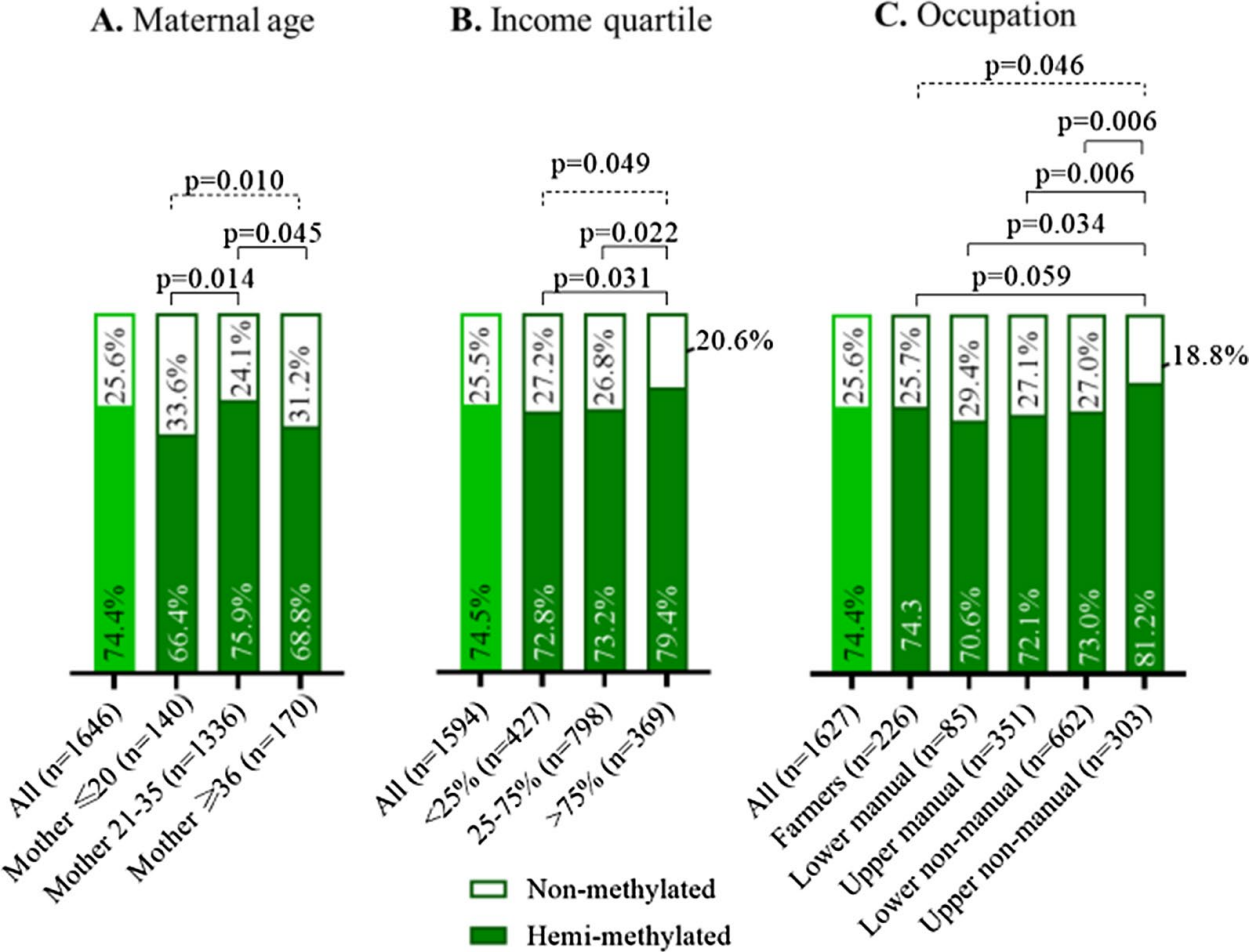
The associations between nc886 epiallele status and early life conditions. The prevalence of *nc886* epiallele methylation status is presented according to **a** mother’s age, **b** family’s income quartile, and **c** family’s occupational status. The white portion of the columns describes the proportion of non-methylated individuals and green the portion that of hemi-methylated individuals. Due to low numbers, intermediately methylated individuals were discarded from the analysis. The prevalence of non-methylated individuals is lower among individuals born to mothers aged 21–35 years (**a**), or to families in the highest income quartile or families with at least one parent working in an upper non-manual occupation. Between-group comparisons analyzed with a Chi-squared test are presented by solid lines and Chi-squared test over groups by dotted lines

Previous reports have indicated that periconceptional conditions associate with the establishment of the nc886 epiallele. Carpenter et al. have shown that children born to young mothers are more likely to present a non-methylated *nc886* methylation status [1, 21, 22]. Similarly, in the YFS, we show that individuals born to younger mothers (≤ 20 years) and also those born to older mothers (≥ 36 years), where more likely to have non-methylated *nc886* epiallele (Fig. 5a) compared to individuals born to mothers aged 21–35 years (*p* < 0.05). Maternal nutritional status and especially low levels of one-carbon metabolism substrates and co-factors have also been linked to a higher probability of the child being born with a non-methylated *nc886* epiallele [4]. We lacked data on maternal periconceptional nutritional status, but as micronutrient intake and status has been shown to be associated with SES [43], we decided to investigate the associations between the family’s SES during childhood (measured in 1980 when the individuals were aged 3–18 years) and *nc886* epiallele methylation status. In the YFS, we observed that individuals born to families in the highest income quartile or to families with an upper non-manual occupational status had a lower probability to harbor a non-methylated *nc886* epiallele (Fig. 5b, c). However, parental education status did not have an association with *nc886* epiallele methylation status. It should be noted that maternal age is associated with SES [44], which can also be observed in the YFS, where both young and older mothers are underrepresented in the highest income and occupational status groups.

These findings are in line with the hypothesis that the substrates and cofactors of one-carbon metabolism during the periconceptional period could affect the imprinting process of the *nc886* epiallele [20, 45]. In mice, it has been experimentally established that the restriction and supplementation of folate can regulate the methylation status of imprinted genes [46].

### Methylation status of the *nc886* epiallele associates with metabolic traits in childhood and adolescence

**Fig. 6:**
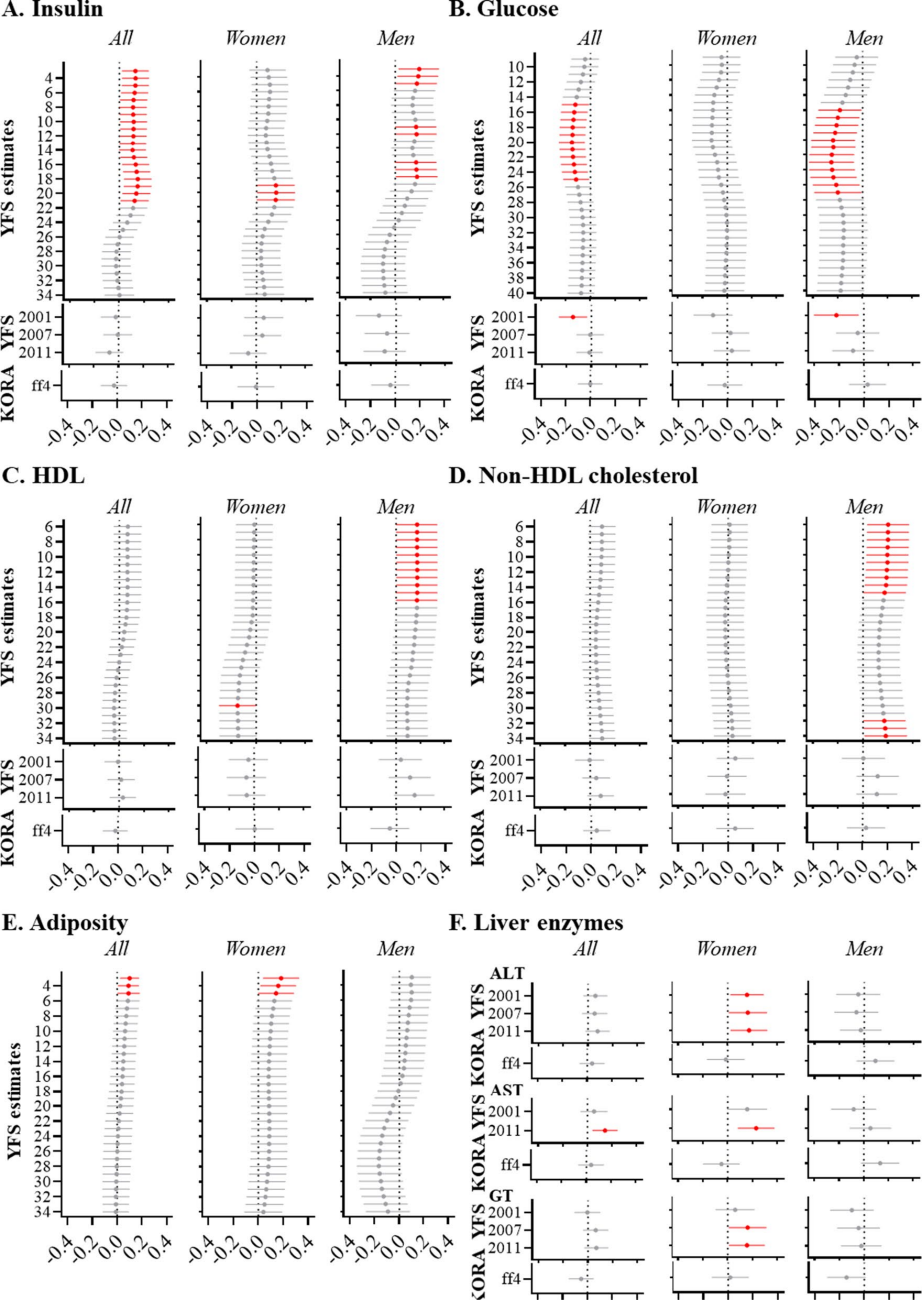
*nc886* epiallele methylation and metabolic traits. Associations between *nc886* epiallele methylation status and yearly estimates (from ages 6–34) and measurements of **a** insulin, **b** glucose, **c** HDL cholesterol, **d** non-HDL cholesterol, **e** adiposity, and **f** liver enzymes. For adiposity, only yearly estimates from the YFS are available, while only biochemical measurements are available for liver enzymes. Values are expressed as standard deviation (SD) increments in metabolite measures and as 95% confidence intervals (95% CI) comparing individuals with a non-methylated *nc886* epiallele to those with a hemi-methylated epiallele. Intermediately methylated individuals were excluded from the analysis due to low numbers. Nominal *p* value < 0.05 is indicated with red

Imprinted genes are widely associated with fetal growth and metabolic traits in later life [30, 31]. Therefore, we set out to investigate the associations of *nc886* methylation status with an individual’s health traits and metabolic measurements. For the YFS, we had information about the health traits measured at regular intervals from 1980 until 2011. From these measurements, yearly estimates of these health-related traits and AUCs describing the levels of the traits during the age period were generated. The associations between the *nc886* epiallele methylation status (non- vs. hemi-methylated) and the yearly estimates can be seen in Fig. 6 (*n* = 1654).

Individuals with a non-methylated *nc886* epiallele had higher (*p* < 0.05) estimated insulin levels during early life (ages 6–24) in comparison with those with a hemi-methylated epiallele (Fig. 6a, Additional file 1: Table S11). Correspondingly, these individuals also had lower serum glucose levels during early life, more significantly during young adulthood (ages 18–24) (Fig. 6b, Additional file 1: Table S11). This association with glucose was also seen in the YFS 2001 follow-up measurements (Fig. 6b). Also, the estimated adiposity for ages 4–6 was higher among the individuals with a non-methylated *nc886* epiallele in comparison with those with a hemi-methylated epiallele (Fig. 6e). This is supported, to a degree, by the previous finding of a non-methylated status of the *nc886* epiallele being associated with higher BMI in early childhood [24], even though this specific association was not replicated in YFS.

In a sex-stratified analysis, men with a non-methylated *nc886* epiallele had higher estimated insulin levels in childhood and adolescence (ages 6–18) and lower glucose levels in young adulthood and early life in comparison with those with a hemi-methylated *nc886* epiallele (Fig. 6a, b, Additional file 1: Table S11). They also had higher estimated HDL and non-HDL cholesterol levels during childhood (ages 6–12) and adolescence in the same setting (Fig. 6c, d, Additional file 1: Table S11). Women with a non-methylated *nc886* epiallele also had higher estimated insulin levels in young adulthood and early life in comparison with those with a hemi-methylated *nc886* epiallele (Fig. 6a, Additional file 1: Table S11). In addition, liver enzyme levels were elevated in women in a similar setting in the 2001, 2007, and 2011 follow-ups (Fig. 6f).

In addition to the liver enzyme levels in women, no significant results were seen in the later follow-ups (2007 or 2011), and none of them were replicated in KORA FF4, the study featuring older population. Similarly, in the yearly estimates of the YFS, the associations between *nc886* epiallele methylation and metabolic traits can mostly be seen in ages below 25 years, and only non-HDL cholesterol levels in men were associated with the methylation status at an age of over 30 years (Fig. 6d).

The *nc886* epiallele methylation status was not associated with the presence of T2D, impaired fasting glucose (IFG), impaired glucose tolerance (IGT), or prediabetes (either IFG or IGT) in the YFS or KORA cohort. Non-methylated and intermediately methylated individuals were overrepresented in the subpopulation of type 1 diabetics in both cohorts, but the number of type 1 diabetics in these cohorts is too low for a reliable analysis (9 in the YSF and 5 in the KORA) (Additional file 1: Table S12).

These results thus associate, for the first time, the *nc886* epiallele status with metabolic traits in childhood and early adulthood. We hypothesize that later in life, lifestyle factors become more significant contributors to metabolic traits and the development of metabolic diseases than *nc886* epiallele methylation status. However, as *nc886* methylation status remains stable throughout life, we cannot exclude the possibility that this epiallele and the nc886 RNAs also modulate the metabolic traits in late adulthood.

### Serum nc886-3p RNA levels associate with glucose levels and the presence of IFG

To elucidate the connection between *nc886* methylation status and metabolic traits, we analyzed the associations between these traits and the nc886 RNA levels in blood and serum that are regulated by this epiallele. RNA levels of nc886-3p associated (*p* < 0.05) with glucose levels in serum in two follow-ups 25 years apart, and in the 2011 follow-up, we also detected a difference in the level of nc886-3p between individuals with or without IFG in both blood and serum. For nc886-5p, we only detected an association with glucose in serum in the 1986 follow-up, when the participants were aged 3–24 years, but there was also a difference in the serum RNA levels of nc886-5p between IFG and normoglycemic individuals in the 2011 follow-up (Additional file 1: Table S13). Therefore, even though we cannot see an association between nc886 epiallele methylation status and later-life heath traits, we can still observe associations between nc886 RNA levels and indicators of glucose metabolism later in life.

### The *nc886* epiallele is a potential molecular mediator of the DOHaD hypothesis

The imprinting of genes has been hypothesized to develop in response to a parent–offspring conflict, where the maternal and paternal genomes differ in their interests regarding the supply of resources [47]. Paternal genomes favor the opportunistic strategy to enhance the growth of the developing offspring through the expression of growth-enhancing genes, while maternal genomes aim to conserve maternal resources over multiple pregnancies [31]. The physiological function of nc886 RNAs remains largely unknown, although it has been suggested to function as a tumor-sensing molecule [3], oncogene [15], and tumor suppressor [41]. Notably, cancer initiation and progression are associated with the dysregulation of developmental pathways [48–50]. Our results connect *nc886* epiallele methylation and RNA expression, for the first time, to metabolic traits in childhood and early adulthood. We also observed an association between early life adiposity and *nc886* epiallele status, linking this epiallele with early growth. As we and others report that the establishment of *nc886* epiallele methylation is associated with the surrounding conditions during gestation, the *nc886* can be hypothesized to function as a molecular mediator between early-life exposures and later-life health traits, in line with DOHaD/Barker hypothesis [33].

## Limitations of the study

The strength of this study is the availability of multi-omic data from the YFS, as well as the possibility to replicate our findings in the KORA cohort. A further strength is our validation using an iPSC-model. As all studies, ours has limitations. We cannot show causality, as our results comprise several types of association studies. More detailed periconceptional information is needed to identify the conditions affecting the establishment of *nc886* epiallele imprinting. Although we observed an association between metabolic traits and *nc886* epiallele methylation status during adolescence, these effects are possibly altered by lifestyle factors as individuals age and thus cannot be replicated in the KORA study, which consists of older individuals. Furthermore, the two iPSC lines exhibiting the highest levels of nc886 RNA expression were originally derived from diabetic individuals, which could affect the results. Finally, in this research paper, we have analyzed nc886-3p and nc886-5p as individual molecules with the assumption that they function as or akin to miRNAs. This is currently being discussed in the literature; further discoveries could change the interpretation of our findings.

## Conclusions

We show how *nc886* methylation fits into the definition of a metastable epiallele and elucidate the implementation of its effects through nc886 RNA expression. Firstly, we demonstrate that the methylation of the *nc886* epiallele is a probabilistic event, the probability of which is associated with early life conditions. After establishment, the methylation pattern remains stable in blood, at least during the 25-year follow-up. The nc886 epiallele methylation regulates the expression of nc886 RNAs coded within its boundaries, and both the methylation status and the expression of the nc886 RNAs associate with metabolic traits, especially the indicators of glucose metabolism. This, in turn, is in line with the general tendency of imprinted genes to associate with metabolic traits after gestation. Taken together, our results suggest that *nc886* is a potential molecular mediator between early life exposures and health traits in later life.

## Methods

More detailed methods can be found in Additional file 1.

### Cohort/sample description

The YFS is a multicenter follow-up study on cardiovascular risk from childhood to adulthood in Finland [51]. The YFS was launched in 1980, with 3596 children and adolescents (aged 3–18 years) participating. Thereafter, the participants have been followed with several examinations, including comprehensive risk factor assessments with major follow-ups in 1986, 2001, 2007, and 2011 (Additional file 1: Figure S1A).

The KORA studies comprise a series of population-based epidemiological surveys and follow-up examinations of individuals living in the region of Augsburg and two adjacent counties in Southern Germany [52]. The analyses included in the present study were based on the KORA-F4 (2006/2007, *n* = 3080) and FF4 (2013/2014, *n* = 2279) studies, both follow-up studies of the KORA Survey S4, conducted in 1999/2001 (*n* = 4261) (Additional file 1: Figure S1B).

The iPSC lines were produced and maintained as published previously [53]. A total of six iPSC lines were used in this study, and the lines were cultured and characterized as previously described [54]. The iPSCs were differentiated into hepatocytes by a protocol originally described by Kajiwara et al. [55], with slight modifications.

### DNA methylation profiling

From the YFS, genome-wide DNA methylation levels were obtained using the Illumina Infinium MethylationEPIC BeadChip or the Illumina Infinium HumanMethylation450 BeadChip, following the protocol by Illumina. DNA methylation profiling was successful for 312 individuals from the 1986 follow-up and for 1,714 from the 2011 follow-up, with 309 samples overlapping. From the KORA cohort, DNA methylation profiling was successful for 1,727 individuals of the F4 study and for 1,888 of the FF4 study, with 988 individuals overlapping. Methylation was assessed with the Illumina Infinium HumanMethylation450 BeadChip for F4 [56, 57] and with the Illumina Infinium MethylationEPIC BeadChip array for FF4. The epigenetic status of the 6 iPSC lines from d0 (iPSCs) and d19 (hepatocytes) and of 12 samples from the YFS cohort was assed using Agena’s Epityper method and the MassArray Analyzer Compact Maldi-TOF-instrument.

### RNA isolation and sncRNA arrays

Whole blood from YFS participants was collected into PAXgene tubes and RNA from blood; serum and iPSC samples were isolated with appropriate methods. Short non-coding RNA expression profiling was performed with the TaqMan® OpenArray® MicroRNA Panel, also containing nc886-3p and -5p, using the AccuFill System and run with the QuantStudio 12K Flex.

### Gene expression profiling

The genome-wide gene expression profiling of the YFS blood samples from 2011 was performed with an Illumina HumanHT-12 version 4 Expression BeadChip, as described earlier [58, 59], utilizing the same RNA sample for both mRNA and sncRNA expression profiling.

The nc886-102nt was separately quantified with qRT-PCR from the serum and blood of 50 individuals from the YFS and from the 6 iPS cell lines from d0. The nc886-102nt was quantified with an Applied Biosystems assay for vtRNA2-1 (Hs04273370_s1), and B2M (Hs00187842_m1) was utilized as a reference gene.

### Genome-wide genotyping from the YFS

Genotyping was performed using a custom-built Illumina Human 670 k BeadChip at the Welcome Trust Sanger Institute [60]. Genotypes were called using the Illuminus clustering algorithm. After quality control, 546,677 genotyped SNPs were available for further analysis. Genotype imputation was performed using Minimac3 [61] and the 1000G phase3 reference set on the Michigan Imputation Server. Autosomes and sex chromosomes were phased using Eagle [62] and SHAPEIT [63], respectively.

### Clinical and biochemical measurements

In the YFS, weight and height were measured, and BMI was calculated. Glucose, cholesterol, HDL, LDL, insulin, glycated hemoglobin triglyceride, ALT, AST, and GT concentrations were measured with standard methods, as described previously [58]. LDL and non-HDL cholesterol levels were calculated. Individuals were categorized into the normoglycemic (NG), IFG, and T2D groups according to the WHO criteria [64].

The repeatedly measured data from the YFS were leveraged to describe long-term trends in weight, height, BMI, adiposity, as well as serum glucose, insulin, and lipid levels were utilized to calculate the area under the curve (AUC) separately for each variable [65]. Individual AUCs were then utilized to create estimate values for measurements for each age period from childhood to adulthood. The AUC variables were defined separately for childhood (6–12 years), childhood and adolescence (6–18 years), adolescence (12–18 years), young adulthood (18–24 years), and early life (6–24 years).

The KORA FF4 study participants underwent an extensive standardized medical examination, including the collection of blood samples. Weight and height were measured, and BMI was calculated [66, 67]. Glucose, HDL and LDL cholesterol, triglyceride, HbA1c [68], serum insulin [69], GT, AST, and ALT [70] levels were measured by standardized methods, and non-HDL cholesterol levels were calculated. All KORA FF4 participants without known diabetes were assigned to receive a standard 75 g oral glucose tolerance test (OGTT) as described previously [68]. Individuals were categorized into the normoglycemic (NG), IFG, IGT, and T2D groups according to WHO criteria [64]. Previously known type 2 diabetes was defined as a self-report that could be validated by the responsible physician or a medical records review, or as current use of glucose-lowering medication.

### Birth family socioeconomic factors and maternal age at birth in the YFS

Maternal age at childbirth was calculated by subtracting the age of the YFS participant in 1980 from the reported age of the mother at the same follow-up. Individuals were divided into groups according to maternal age at childbirth: ≤ 20, 21–35, and ≥ 36. The occupation of the parents was obtained by a questionnaire in 1980 and classified as I, upper non-manual; II, lower non-manual; III, upper manual; IV, lower manual; and F, farmers [71]. The parents’ education in 1980 was determined on the basis of school years completed, and it was classified into three groups: < 9 years (I), 9–12 years (II), and > 12 years (III). The information of the parent with the most years of schooling was used in the study [72]. Family income was divided into three groups: (I) lowest quartile, (II) interquartile range, and (III) the highest quartile.

### Statistical analysis

An EWAS for whole blood RNA levels of nc886-3p and -5p separately was performed with appropriate adjusting variables for the YFS samples profiled with an EPIC array (*n* = 1526).

A clustering of samples based on *nc886* methylation was performed separately for samples profiled with Illumina 450 K and EPIC arrays, separately for follow-up studies, and separately for different cell types of GSE131989 [35]. The clustering was based on 14 CpG sites, located within 2000 bp around the coding region of *nc886*, that showed a bimodal distribution of methylation values (Additional file 1: Figure S2) and was performed using hierarchical clustering (R, hclust with default settings). The stability of the methylation status was evaluated with the individuals from the YFS and KORA with repeated DNA methylation profiling available. Median methylation values for the 14 CpG sites selected for the clustering of individuals were calculated and compared between follow-ups.

The nc886 RNA expression data was analyzed with the ΔΔ*Cq* method. Fold changes for nc886-3p, -5p, and -102nt were calculated separately for the ncRNAs in the YFS blood and serum subpopulations, using the median of the hemi-methylated group as the reference. For iPSCs, the median of all samples at d0 was used as the reference value. Between-group differences were evaluated with the Mann–Whitney U test and trend over status groups with the Kruskal–Wallis test.

Genome-wide association analyses on *nc886* methylation status and on miRNA expression levels were performed using regression models in SNPTEST v2.5.4. A GWAS for methylation status (non- vs hemi-methylated) was performed with a logistic regression model adjusted with appropriate variables. In addition, the GWAS results on the 18 CpG sites located in the *nc886* DMR were retrieved from the GoDMC database (http://mqtldb.godmc.org.uk/index.php) [73], and an association analysis in the YFS data was performed for these 18 CpG sites and genetic variations located ± 1 Mb of *nc886* loci. Also a similar GWAS for nc886-3p and nc886-5p each was performed.

The association of the parental income, education, occupational status, and maternal age groups with the prevalence of hemi- and non-methylated individuals was assessed by comparing the groups one by one with the Chi-squared test.

A differential gene expression analysis of genome-wide gene expression data with respect to *nc886* methylation status (non- vs. hemi-methylated) was performed using limma and Biobase R/Bioconductor packages. The analysis was adjusted with appropriate variables. A gene set analysis of the gene expression data with respect to the two *nc886* methylation categories was carried out with curated gene sets downloaded from the Molecular Signature Database (MSigDB) on April 8, 2020 using a threshold-free gene set analysis method, mGSZ [74].

For the target mRNA–driven GSEA for nc886-3p and -5p RNA levels, the predicted mRNA targets of nc886-3p and nc886-5p were included in the correlation analysis if they were recognized by microRNA.org. Correlations between nc886-3p and -5 and their predicted targets were calculated, and individual correlations with FDR < 0.05 were considered significant. Predicted target mRNAs that correlated at the level of *p* < 0.05 with the RNA levels of nc886-3p or -5p were selected for further pathway enrichment analyses. The overlaps between the selected target mRNAs and gene set in KEGG and BIOCARTA were analyzed in a molecular signature database. Gene sets containing at least 5 of the selected target mRNAs (FDR < 0.05) were considered to be enriched with the targets that correlated with the expression of the ncRNA. The analysis was first run separately for genes correlating with nc886-3p and -5p, and then together with all target mRNAs correlating with at least one of the ncRNAs.

Associations between the *nc886* epiallele methylation status and the YFS yearly estimates of metabolic phenotypes was assessed one by one with adjusted linear regression model. The analysis was repeated separately with men and women. Similar regression model was used to analyze the association of *nc886* epiallele methylation status and metabolic measures from the YFS 2001, 2007, and 2011 follow-ups and KORA FF4.

The association between the blood and serum nc886-3p and -5p measurements from 1986, 2001, and 2011 and the metabolic traits measured during the same follow-up was analyzed with adjusted linear regression models. Similarly, the association between impaired fasting glucose and nc886-3p and -5p RNA levels in 2011 was analyzed with an adjusted linear regression model predicting nc886 RNA levels with the glycemic status (impaired fasting glucose yes/no 2011).

## Supplementary Information

**Additional file 1.** Supplementary Figures S1-6, Supplementary Tables S1-3 and S11-13 and Supplementary materials and methods.

**Additional file 2.** Supplementary Tables S4-10.

## Data Availability

The datasets generated and/or analyzed during the current study regarding YFS and KORA are not publicly available due to restrictions imposed by Finnish and German legislation but are available from the corresponding author/data sharing committees upon a reasonable request. GSE131989 (GEO; https://www.ncbi.nlm.nih.gov/geo/query/acc.cgi?acc=GSE131989) and data available in http://mqtldb.godmc.org.uk/index.php were also utilized in this manuscript and are publicly available.
