# Supplementary material for: Methylation status of nc886 epiallele reflects periconceptional conditions and is associated with glucose metabolism through nc886 RNAs
Source: Clin Epigenetics. 2021 Jul 22;13:143. doi: 10.1186/s13148-021-01132-3 (PMC8296652; doi:10.1186/s13148-021-01132-3)
Supplement: Supplementary file 1 — Additional file 1. Supplementary Figures S1-6, Supplementary Tables S1-3 and S11-13 and Supplementary materials and methods. [file 13148_2021_1132_MOESM1_ESM.docx]

**Methylation status of the *nc886* epiallele reflects periconceptional conditions and is associated with glucose metabolism through the nc886 RNAs**

Saara Marttila, Leena E. Viiri, Pashupati P. Mishra, Brigitte Kühnel, Pamela R. Matias-Garcia, Leo-Pekka Lyytikäinen, Tiina Ceder, Nina Mononen, Wolfgang Rathmann, Juliane Winkelmann, Annette Peters, Mika Kähönen, Nina Hutri-Kähönen, Markus Juonala, Katriina Aalto-Setälä, Olli Raitakari, Terho Lehtimäki, Melanie Waldenberger, Emma Raitoharju

**Corresponding Authors:** Emma Raitoharju, Tampere University, Finland. E-mail: emma.raitoharju@tuni.fi and Saara Marttila, Tampere University, Finland. E-mail: saara.marttila@tuni.fi

Contents

[Additional file figures 2](#_Toc66267452)

[Additional file 1: Figure S1. 2](#_Toc66267453)

[Additional file 1: Figure S2. 3](#_Toc66267454)

[Additional file 1: Figure S3. 5](#_Toc66267455)

[Additional file 1: Figure S4. 6](#_Toc66267456)

[Additional file 1: Figure S5. 7](#_Toc66267457)

[Additional file 1: Figure S6. 8](#_Toc66267458)

[Additional file 1: tables 11](#_Toc66267459)

[Additional file 1: Table S1. 11](#_Toc66267460)

[Additional file 1: Table S2 12](#_Toc66267461)

[Additional file 1: Table S3. 13](#_Toc66267462)

[Additional file 1: Tables S4–S10 can be found in Additional file 1: file 2. 13](#_Toc66267463)

[Additional file 1: Table S11. 14](#_Toc66267464)

[Additional file 1: Table S12. 15](#_Toc66267465)

[Additional file 1: Table S13. 16](#_Toc66267466)

[Additional file 1: materials and methods 17](#_Toc66267467)

[Cohort/sample description 17](#_Toc66267468)

[DNA methylation profiling 20](#_Toc66267469)

[RNA isolation and sncRNA arrays 23](#_Toc66267470)

[Gene expression profiling 24](#_Toc66267471)

[Genome-wide genotyping in the YFS 24](#_Toc66267472)

[Clinical and biochemical measurements 24](#_Toc66267473)

[Birth family’s socioeconomic factors and maternal age at birth in the YFS 26](#_Toc66267474)

[Statistical analysis 26](#_Toc66267475)

[Additional file 1: references 29](#_Toc66267476)

## Additional file 1: figures

Additional file 1: Figure S1. The follow-ups and data utilized from the (A) Young Finns Study (YFS) and (B) the Cooperative Health Research in the Region Augsburg cohort (KORA). For the YFS, the genome-wide association study was performed with samples from 2007 that were supplemented with samples from 2001 of participants who did not participate in the 2007 follow-up.


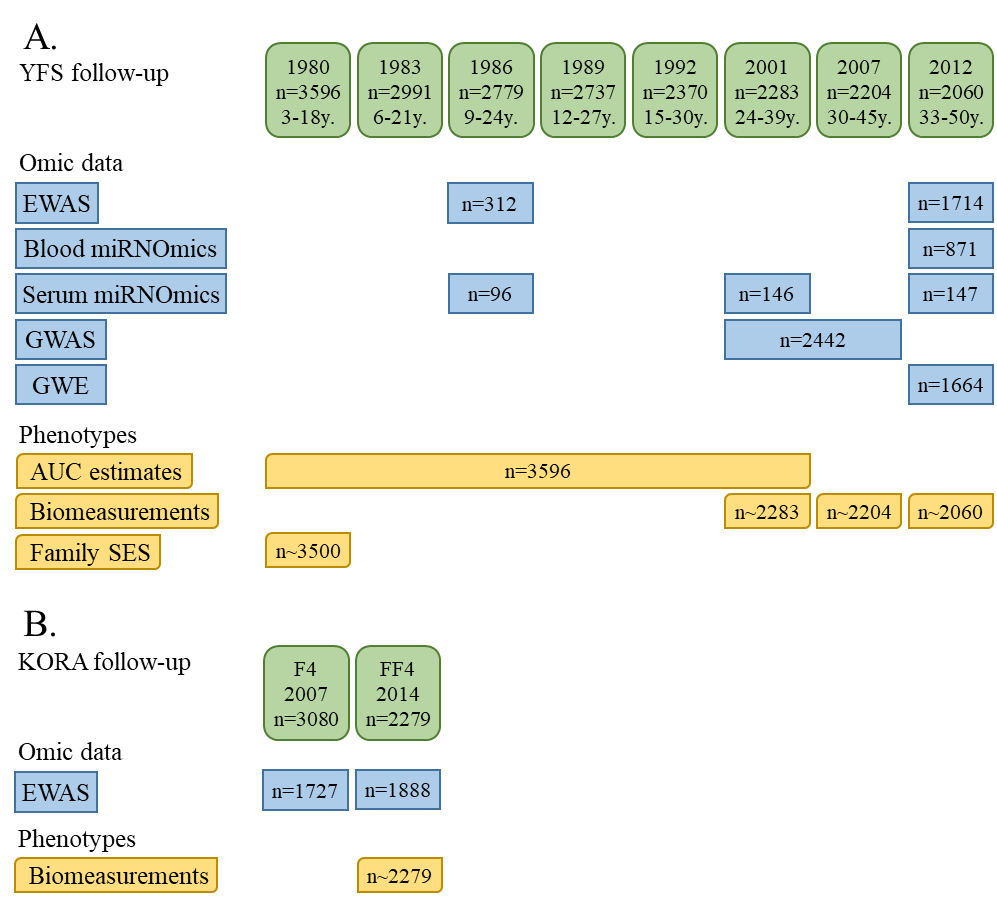


**Abbreviations:** EWAS = epigenome-wide association study; GWAS = genome-wide association study; GWE = genome-wide expression study; AUC estimates = area under the curve estimates for weight, height, BMI, adiposity as well as serum glucose, insulin, and lipids levels; SES = socioeconomic status

Additional file 1: Figure S2. Clustering of individuals based on the methylation level of the *nc886* epiallele. (A) Frequency of the methylation β values at the 18 CpG sites in the *n866* DMR. The 14 CpGs presenting bimodal distribution are indicated in green color. (B) example dendrogram from the hierarchical clustering; and (C) median methylation of the 14 CpG sites with bimodal distribution. In all figures, the samples are from the YFS 2011 follow-up analyzed with the EPIC array (n = 1,526).


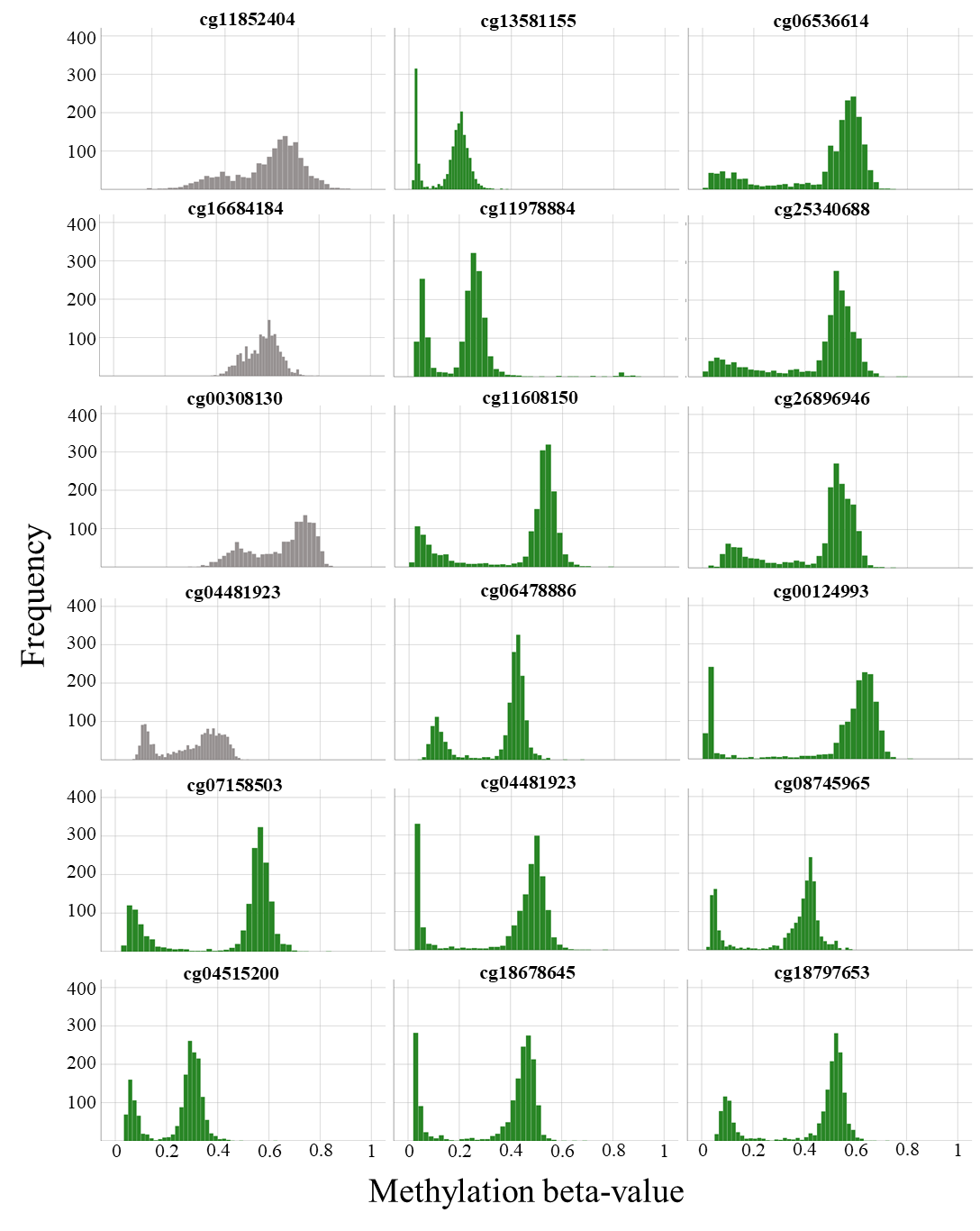


**A.**


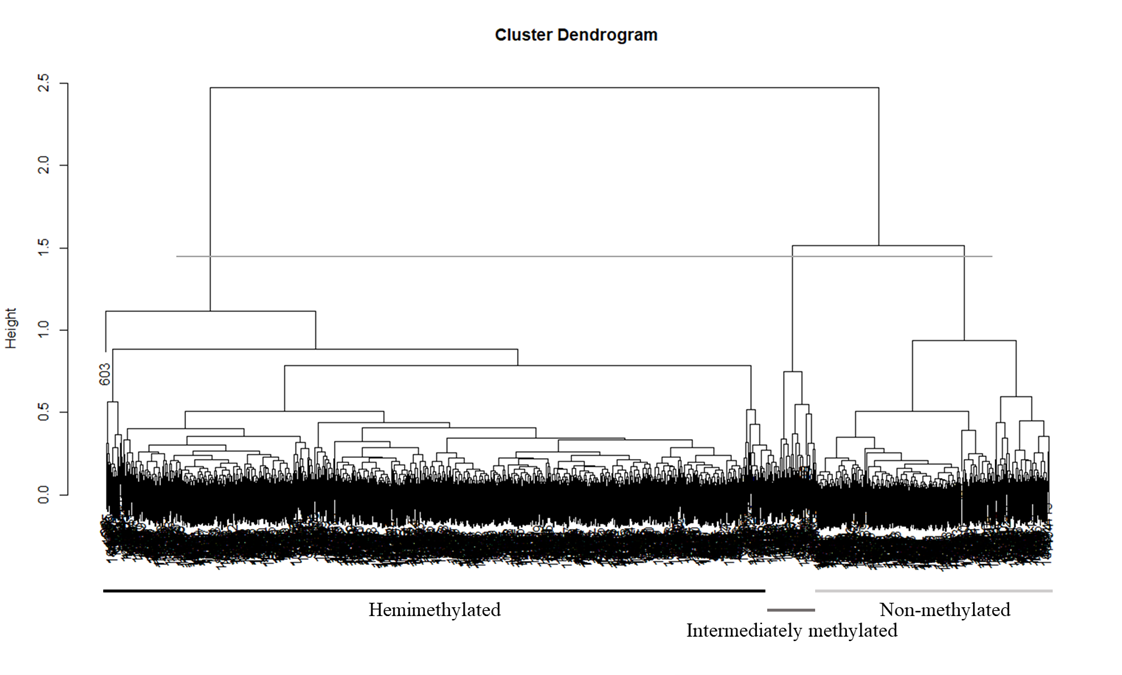


**B.**


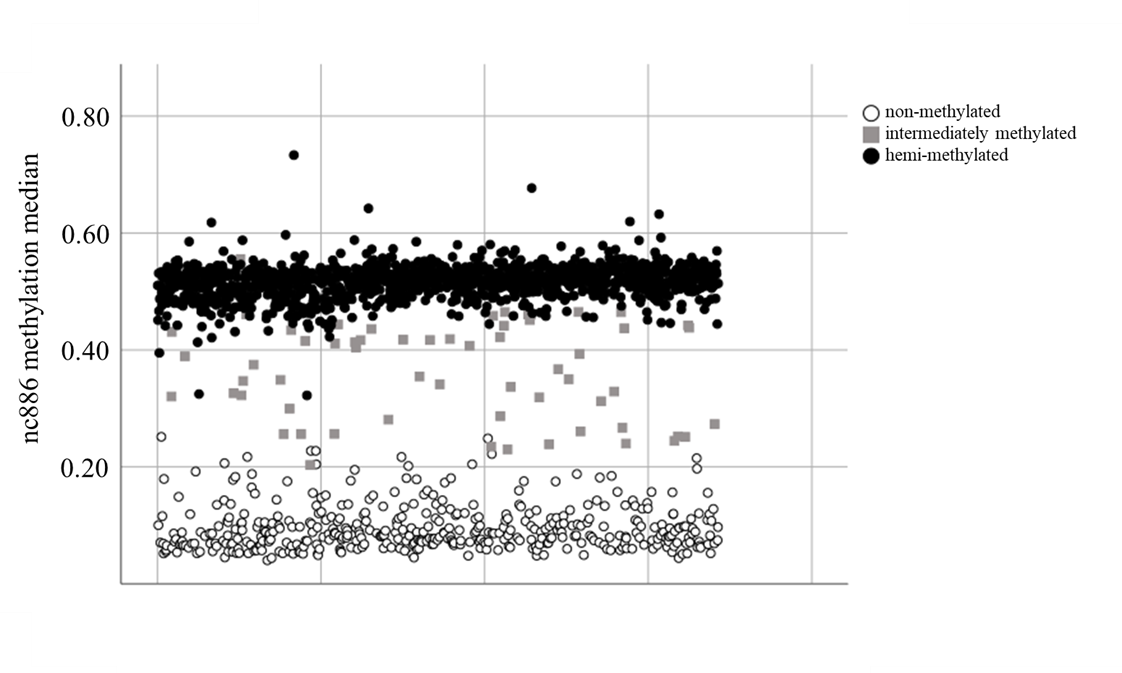


**C.**

Additional file 1: Figure S3. Comparison of the median *nc886* DMR methylation levels of the 12 YFS participants (2011 follow-up) with DNA methylation array and mass spectrometry–based bisulfite sequencing (EpiTyper) data available. According to the DNA methylation array clustering, three individuals clustered into the non-methylated group (white), six into the intermediately methylated group (light gray), and three into the hemi-methylated group (dark gray).

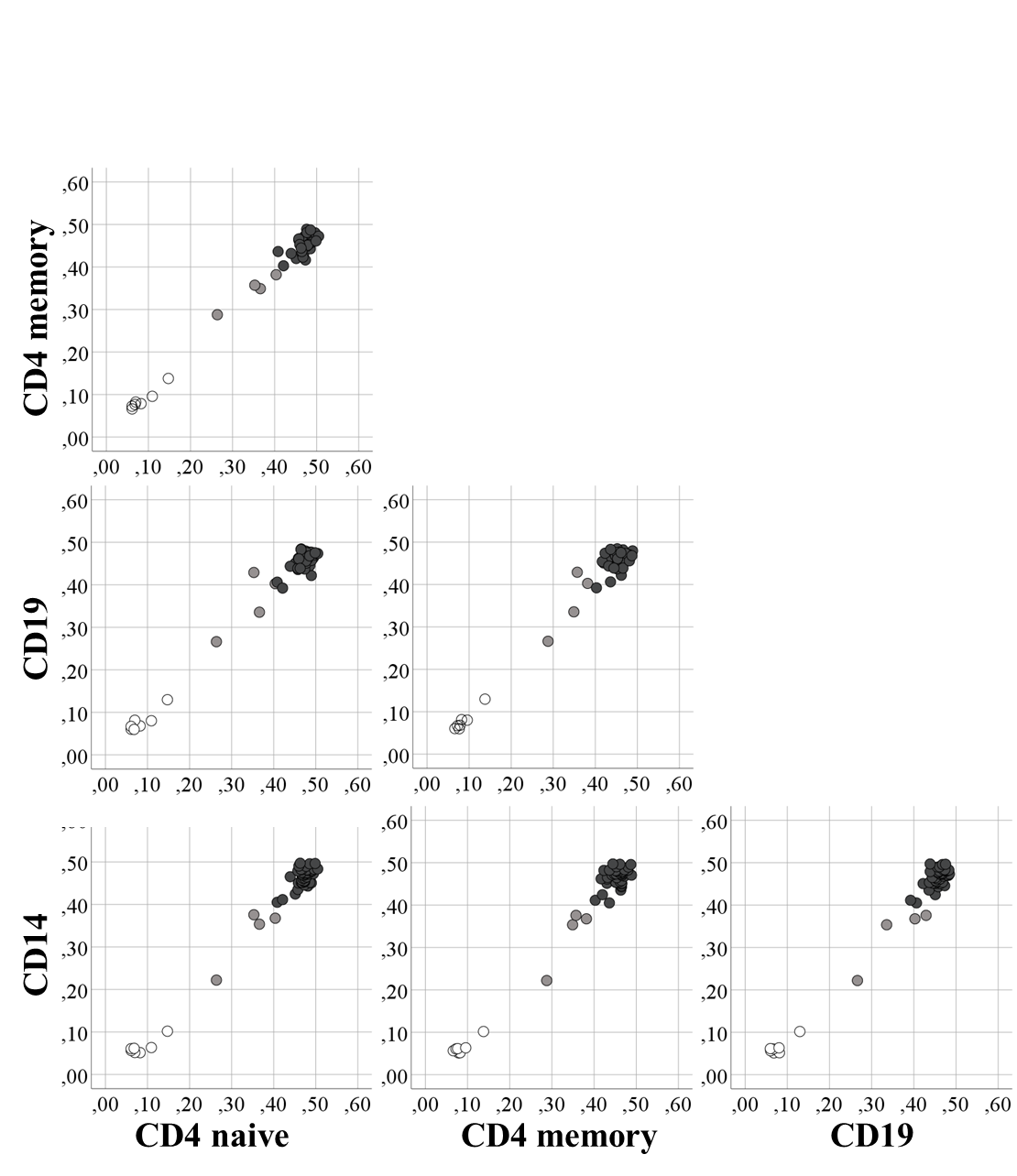
Additional file 1: Figure S4. Median methylation of the 14 CpG sites at the *nc886* epiallele in monocytes (CD14+), B-cells (CD19+), and naïve or memory T cells (CD4 naïve or CD4 memory) from the data of Rhead et al. ^1^ (GSE131989). Figures present the median methylation β values of different cell populations from 59 individuals who could be reliably mapped as specific individuals according to the phenotype data available. Individual dots are colored according to the *nc886* epiallele methylation group that the individual mostly clustered into (non-methylated = white, intermediately methylated = light gray, hemi-methylated = dark gray). Data indicates that the methylation status of the *nc886* epiallele is very similar in different blood cell populations and that the intermediately methylated status is not caused by a different methylation status being present in different blood cell types.

Additional file 1: Figure S5. Comparison of nc886-102nt, -3p, and -5p levels in the (A) blood and (B) serum of 50 YFS participants from the 2011 follow-up, and in (C) iPSCs (d0). Cases have been sorted according to ascending nc886-102nt levels. In A and B, fold changes have been calculated in reference to the median of the hemi-methylated *nc886* epiallele group and, in C, in reference to the median of all measured samples at d0.


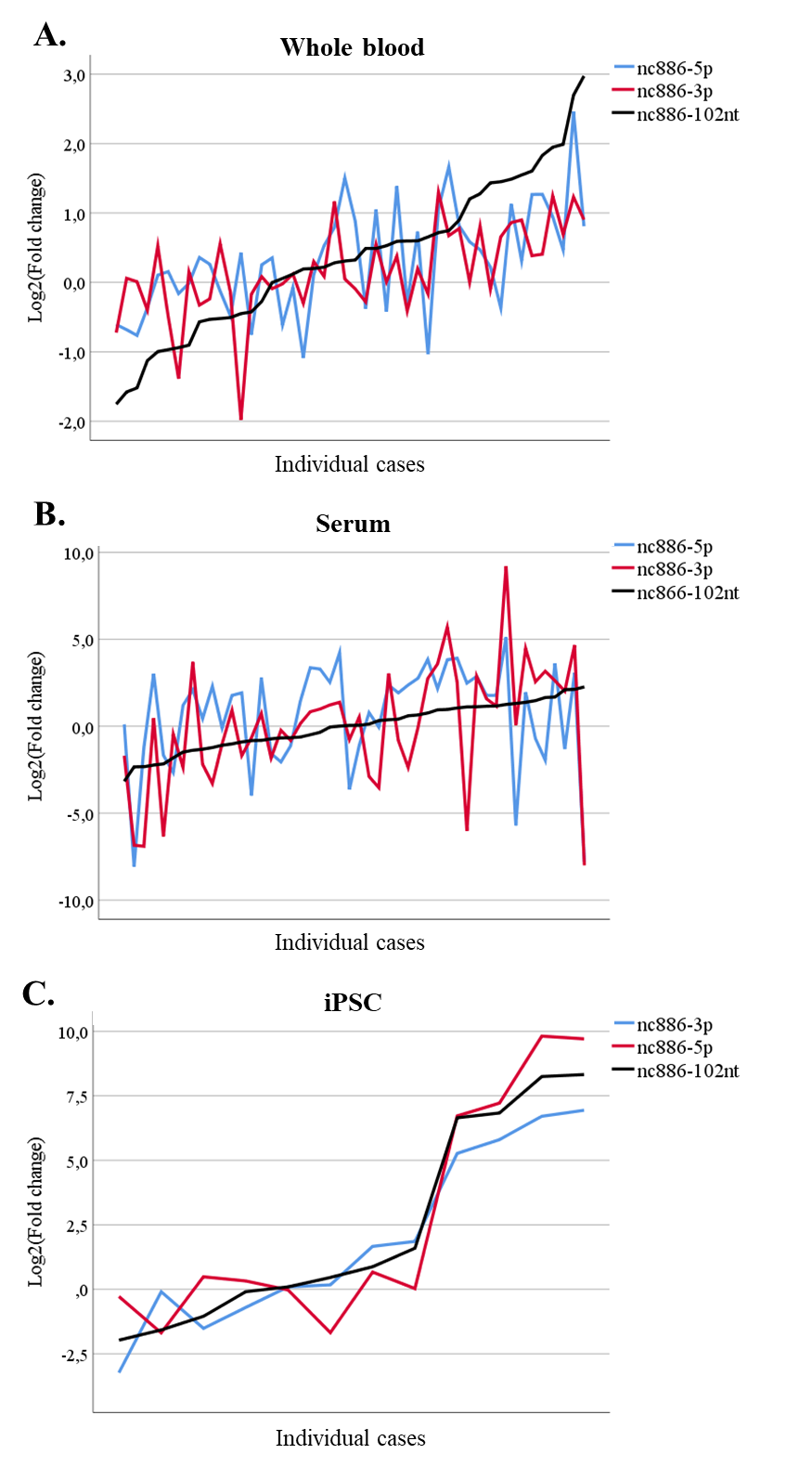


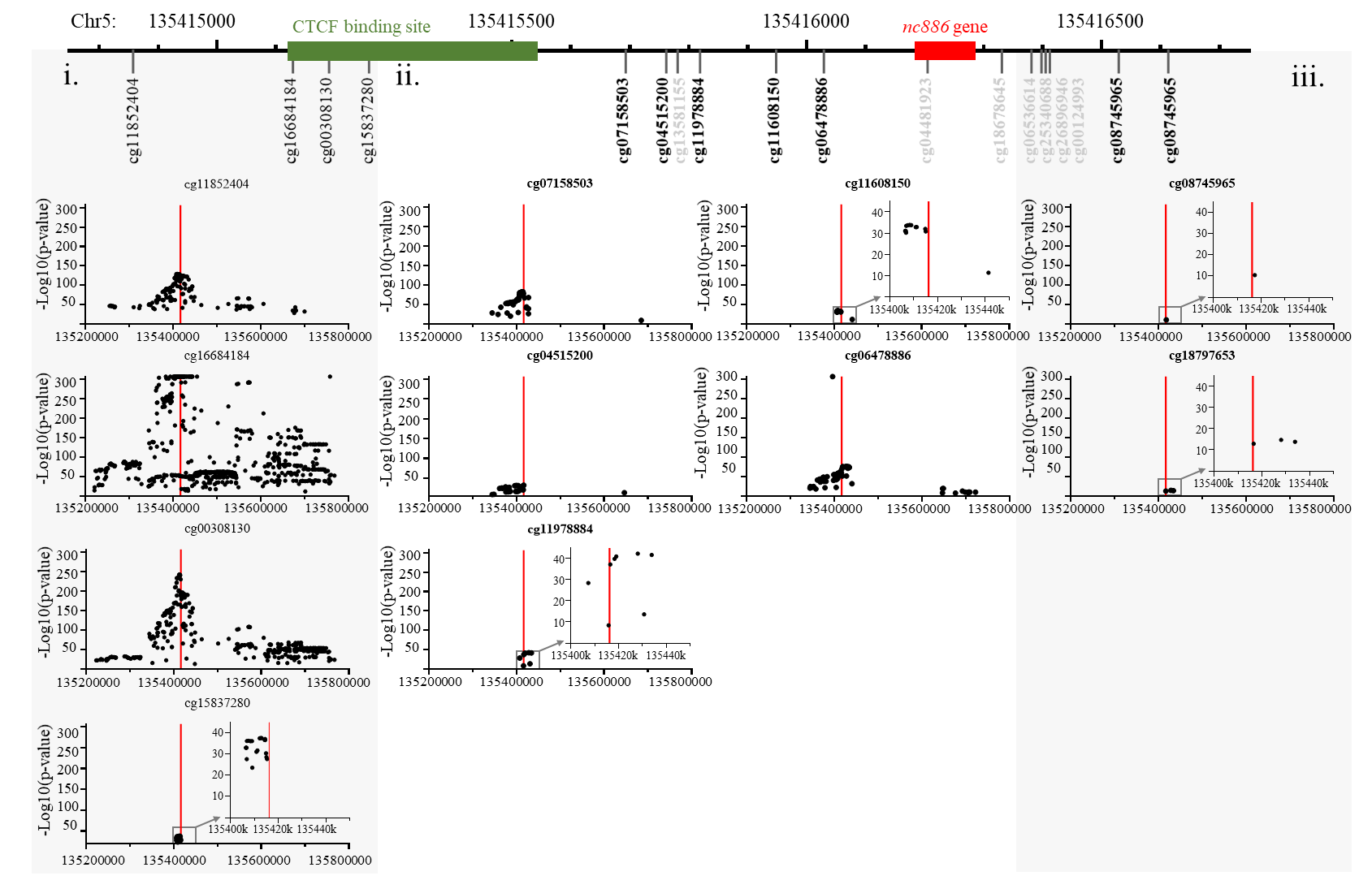


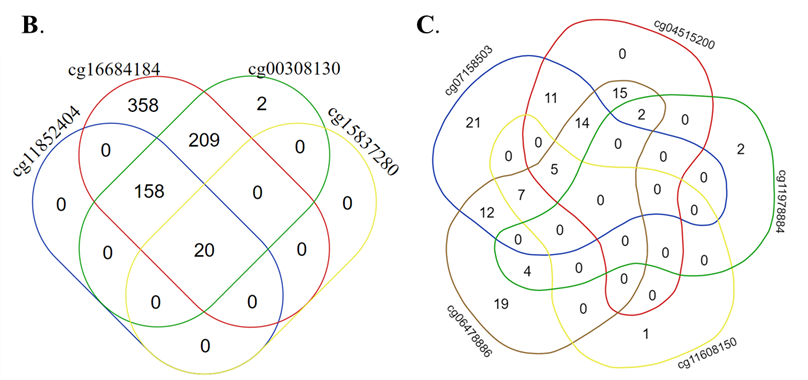


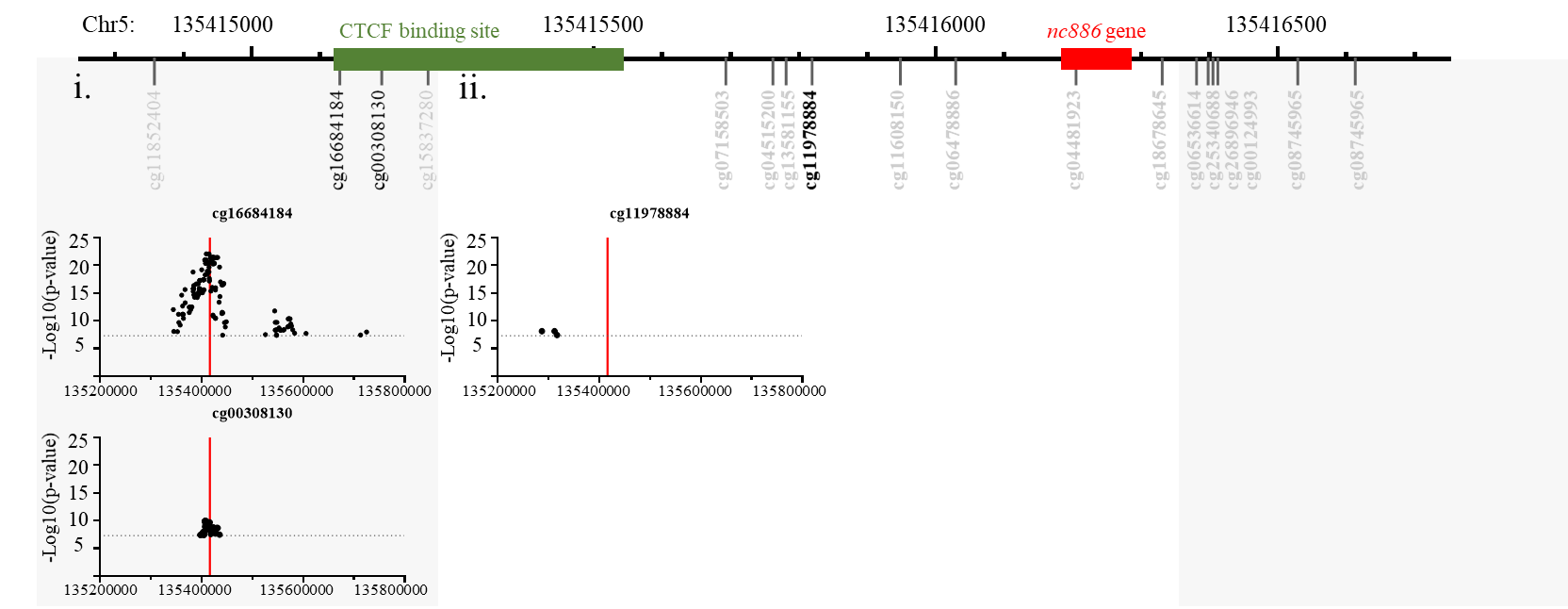


Additional file 1: Figure S6. Associations between the methylation levels of the 18 CpGs in the nc886 DMR and genetic variation in (A–C) the Genetics of the DNA Methylation Consortium (GoDMC data (http://mqtldb.godmc.org.uk/; n≈32000), and (D) in the YFS 2011 EPIC data (n = 1,526). (A) In the GoDMC data, a multitude of associations are detected with the four GpGs presenting non-bimodal methylation patterns (i, not in boldface) and some with those bimodally distributed CpGs that are located down-stream from the nc886 gene (ii). No association was detected with the 6 CpGs with a bimodal distribution which are located on or directly up-stream from the nc886 gene (indicated with gray front color), and only four associations were identified with the two CpGs located further up-stream from nc886 (iii). (B) The genetic variations that associated with the non-bimodally distributed CpGs (i) overlapped greatly. (C) Far less overlap was seen with those variations that associated with the bimodally distributed CpGs down-stream from nc886 and only 5 correlating SNPs (rs11742191, rs11749522, rs17169806, rs62365993, rs2881285) associated with at least 4 of the 14 bimodally distributed CpGs (cg04515200, cg06478886, cg07158503, cg11608150) (ii). (D) In YFS, only the methylation of cg16684184, cg00308130, and cg06536614 associated with genetic variation. Almost all of the associations with cg16684184 and cg00308130 (with the exception of rs7726617, rs142812848, and rs4572 associating with cg16684184) were also seen in the GoDMC data. In contrast, none of the 3 variations associating with cg06536614 were observed to be associated with the methylation of this CpG in the GoDMC data.

## Additional file 1: tables

Additional file 1: Table S1. Associations between nc886-3p and -5p levels in blood and the genome-wide DNA methylation in the YFS 2011 samples analyzed with EPIC (for nc886-3p n = 806 and for nc886-5p n = 825). CpG sites used to determinate an individual’s methylation status at the *nc886* epiallele are presented in boldface, and the GpC sites outside the 1.9-kb *nc886* DMR are italicized. Associations have been analyzed with the ΔC_T_ values, and the beta value from the regression model is thus inversely associated with the concentrations of the sncRNA.

|  | **probeID** | **BETA** | **SE** | **P_VAL** | **chr** | **pos** | **strand** | **UCSC RefGene Name** | **UCSC RefGene Group** |
| --- | --- | --- | --- | --- | --- | --- | --- | --- | --- |
| **nc886-3p** | |  |  |  |  |  |  |  |  |
|  | *cg19031085* | -3.491 | 0.317 | 6.87*10^-26^ | chr5 | 135228288 | - | IL9 | Body |
|  | **cg06478886** | 2.312 | 0.214 | 3.81*10^-25^ | chr5 | 135416029 | - |  |  |
|  | **cg04515200** | 2.864 | 0.267 | 9.43*10^-25^ | chr5 | 135415762 | + |  |  |
|  | **cg18797653** | 1.687 | 0.158 | 1.26*10^-24^ | chr5 | 135416613 | + | MIR886 | TSS1500 |
|  | **cg07158503** | 1.468 | 0.138 | 2.83*10^-24^ | chr5 | 135415693 | - |  |  |
|  | **cg18678645** | 1.627 | 0.157 | 2.06*10^-23^ | chr5 | 135416331 | - | MIR886 | TSS200 |
|  | **cg11608150** | 1.545 | 0.149 | 2.09*10^-23^ | chr5 | 135415948 | - |  |  |
|  | **cg04481923** | 1.515 | 0.147 | 3.66*10^-23^ | chr5 | 135416205 | - | MIR886 | Body |
|  | **cg13581155** | 3.719 | 0.364 | 8.53*10^-23^ | chr5 | 135415781 | + |  |  |
|  | **cg00124993** | 1.191 | 0.120 | 8.27*10^-22^ | chr5 | 135416412 | + | MIR886 | TSS200 |
|  | **cg08745965** | 1.806 | 0.183 | 1.65*10^-21^ | chr5 | 135416529 | + | MIR886 | TSS1500 |
|  | **cg25340688** | 1.603 | 0.163 | 2.16*10^-21^ | chr5 | 135416398 | + | MIR886 | TSS200 |
|  | cg15837280 | 2.370 | 0.245 | 9.82*10^-21^ | chr5 | 135415258 | - |  |  |
|  | **cg26896946** | 1.726 | 0.179 | 1.40*10^-20^ | chr5 | 135416405 | + | MIR886 | TSS200 |
|  | **cg06536614** | 1.576 | 0.164 | 2.10*10^-20^ | chr5 | 135416381 | + | MIR886 | TSS200 |
|  | cg00308130 | 2.294 | 0.253 | 1.37*10^-18^ | chr5 | 135415190 | - |  |  |
|  | *cg10187927* | -5.938 | 0.672 | 9.65*10^-18^ | chr5 | 135364060 | + | TGFBI | TSS1500 |
|  | **cg11978884** | 1.993 | 0.251 | 9.93*10^-15^ | chr5 | 135415819 | + |  |  |
|  | *cg16031250* | -5.566 | 0.778 | 2.42*10^12^ | chr5 | 135365012 | - | TGFBI | Body |
|  | *cg26817546* | -4.967 | 0.815 | 1.88*10^-9^ | chr5 | 135538704 | + |  |  |
|  | cg11852404 | 1.852 | 0.325 | 1.89*10^-8^ | chr5 | 135414858 | - |  |  |
|  |  |  |  |  |  |  |  |  |  |
| **nc886-5p** | |  |  |  |  |  |  |  |  |
|  | **cg04515200** | 4.081 | 0.209 | 1.32*10^-66^ | chr5 | 135415762 | + |  |  |
|  | **cg06478886** | 3.249 | 0.168 | 1.28*10^-65^ | chr5 | 135416029 | - |  |  |
|  | **cg18797653** | 2.363 | 0.125 | 2.02*10^-63^ | chr5 | 135416613 | + | MIR886 | TSS1500 |
|  | **cg07158503** | 2.067 | 0.110 | 3.58*10^-63^ | chr5 | 135415693 | - |  |  |
|  | **cg18678645** | 2.326 | 0.124 | 1.08*10^-62^ | chr5 | 135416331 | - | MIR886 | TSS200 |
|  | **cg11608150** | 2.201 | 0.118 | 3.06*10^-62^ | chr5 | 135415948 | - |  |  |
|  | **cg04481923** | 2.164 | 0.116 | 9.15*10^-62^ | chr5 | 135416205 | - | MIR886 | Body |
|  | **cg13581155** | 5.245 | 0.291 | 8.11*10^-59^ | chr5 | 135415781 | + |  |  |
|  | **cg00124993** | 1.714 | 0.095 | 1.47*10^-58^ | chr5 | 135416412 | + | MIR886 | TSS200 |
|  | **cg25340688** | 2.321 | 0.130 | 4.93*10^-58^ | chr5 | 135416398 | + | MIR886 | TSS200 |
|  | **cg26896946** | 2.534 | 0.143 | 2.67*10^-57^ | chr5 | 135416405 | + | MIR886 | TSS200 |
|  | **cg08745965** | 2.589 | 0.146 | 4.97*10^-57^ | chr5 | 135416529 | + | MIR886 | TSS1500 |
|  | **cg06536614** | 2.298 | 0.132 | 9.66*10^-56^ | chr5 | 135416381 | + | MIR886 | TSS200 |
|  | cg15837280 | 3.291 | 0.201 | 1.49*10^-50^ | chr5 | 135415258 | - |  |  |
|  | cg00308130 | 3.272 | 0.208 | 2.24^-47^ | chr5 | 135415190 | - |  |  |
|  | **cg11978884** | 2.863 | 0.211 | 8.38*10^-37^ | chr5 | 135415819 | + |  |  |
|  | cg11852404 | 3.228 | 0.276 | 1.07*10^-28^ | chr5 | 135414858 | - |  |  |
|  | cg16684184 | 4.438 | 0.444 | 6.77*10^-22^ | chr5 | 135415129 | + |  |  |
|  | *cg19031085* | -2.235 | 0.303 | 5.53*10^-13^ | chr5 | 135228288 | - | IL9 | Body |

Additional file 1: Table S2. Spearman rank order correlation between nc886-3p, -5p, and -102nt levels in blood and serum samples in the (A) Young Finns Study and in (B) the iPSC model.

1. **the YFS**

| Serum | nc886-5p | ρ | 1,000 |  |  |  |  |  |  |  |  |  |
| --- | --- | --- | --- | --- | --- | --- | --- | --- | --- | --- | --- | --- |
|  |  | p-value |  |  |  |  |  |  |  |  |  |  |
|  |  | n | 59 |  |  |  |  |  |  |  |  |  |
|  | nc886-3p | ρ | **0.636** | 1,000 |  |  |  |  |  |  |  |  |
|  |  | p-value | **9.21*10^-5^** |  |  |  |  |  |  |  |  |  |
|  |  | n | **32** | 41 |  |  |  |  |  |  |  |  |
|  | nc886-5p | ρ | -0.164 | -0.089 | 1.000 |  |  |  |  |  |  |  |
|  |  | p-value | 0.281 | 0.659 |  |  |  |  |  |  |  |  |
|  |  | n | 45 | 27 | 102 |  |  |  |  |  |  |  |
|  | nc886-3p | ρ | 0.109 | 0.103 | **0.432** | 1.000 |  |  |  |  |  |  |
|  |  | p-value | 0.657 | 0.703 | **0.011** |  |  |  |  |  |  |  |
|  |  | n | 19 | 16 | **34** | 40 |  |  |  |  |  |  |
|  | nc886-5p | ρ | **0.359** | 0.132 | **0.294** | 0.251 | 1.000 |  |  |  |  |  |
|  |  | p-value | **0.010** | 0.463 | **0.004** | 0.140 |  |  |  |  |  |  |
|  |  | n | **51** | 33 | **93** | 36 | 127 |  |  |  |  |  |
|  | nc886-3p | ρ | 0.133 | -0.039 | **0.282** | 0.285 | **0.620** | 1.000 |  |  |  |  |
|  |  | p-value | 0.499 | 0.874 | **0.049** | 0.224 | **2.14*10^-7^** |  |  |  |  |  |
|  |  | n | 28 | 19 | **49** | 20 | **58** | 64 |  |  |  |  |
|  | nc886-102nt | ρ | 0.075 | -0.335 | -0.186 | 0.116 | 0.229 | **0.522** | 1.000 |  |  |  |
|  |  | p-value | 0.752 | 0.263 | 0.251 | 0.680 | 0.118 | **1.43*10^-4^** |  |  |  |  |
|  |  | n | 20 | 13 | 40 | 15 | 48 | **48** | 48 |  |  |  |
| Blood | nc886-5p | ρ | 0.175 | -0.272 | **0.240** | 0.198 | **0.274** | -0.004 | -0.173 | 1.000 |  |  |
|  |  | p-value | 0.186 | 0.085 | **0.015** | 0.220 | **0.002** | 0.976 | 0.241 |  |  |  |
|  |  | n | 59 | 41 | **102** | 40 | **127** | 64 | 48 | 870 |  |  |
|  | nc886-3p | ρ | 0.154 | -0.247 | **0.264** | 0.292 | **0.207** | 0.011 | -0.119 | **0.636** | 1.000 |  |
|  |  | p-value | 0.247 | 0.124 | **0.008** | 0.072 | **0.021** | 0.933 | 0.419 | **2.10*10^-97^** |  |  |
|  |  | n | 58 | 40 | **101** | 39 | **123** | 63 | 48 | **849** | 850 |  |
|  | nc886-102nt | ρ | 0.190 | -0.273 | 0.220 | 0.430 | **0.406** | 0.196 | -0.099 | **0.584** | **0.625** | 1.000 |
|  |  | p-value | 0.465 | 0.417 | 0.185 | 0.097 | **0.005** | 0.188 | 0.516 | **1.61*10^-5^** | **2.69*10^-6^** |  |
|  |  | n | 17 | 11 | 38 | 16 | **47** | 47 | 45 | **47** | **47** | 47 |
|  |  |  | nc886-5p | nc886-3p | nc886-5p | nc886-3p | nc886-5p | nc886-3p | nc886-102nt | nc886-5p | nc886-3p | nc886-102nt |
|  |  |  | Serum | | | | | | | Blood | | |

**B.** iPSC (d0)

| nc886-5p | ρ | 1 |  |  |
| --- | --- | --- | --- | --- |
|  | p-value | . |  |  |
|  | n | 12 |  |  |
| nc886-3p | ρ | **0.734** | 1 |  |
|  | p-value | **0.007** | . |  |
|  | n | **12** | 12 |  |
| nc886-102nt | ρ | **0.811** | **0.979** | 1 |
|  | p-value | **0.001** | **3.09*10^-8^** | . |
|  | n | **12** | **12** | 12 |
|  |  | nc886-5p | nc886-3p | nc886-102nt |

Additional file 1: Table S3. The methylation levels in the nc886 epiallele of the iPSC lines utilized. DNA methylation was measured with mass spectrometry–based bisulfite sequencing at the iPSC stage (d0) and in differentiated hepatocytes (d19).

| **IPSC ID** | **Case/control** | **iPSCs** | **Hepatocytes** |
| --- | --- | --- | --- |
| UTA.08203.DM | Diabetic | 77.5% | 85.0% |
| UTA.10802.EURCC | Diabetic | 78.0% | 89.0% |
| UTA.08102.DM | Diabetic | 89.0% | 91.0% |
| UTA.10211.EURCC | Control | 89.3% | 94.5% |
| UTA.11201.EURCC | Control | 90.3% | 93.3% |
| UTA.11304.EURCC | Control | 93.3% | 90.7% |

### Tables S4–S10 can be found in additional file 2**.**

|  |  | **All** | | | | |  | **Women** | | | | |  | **Men** | | | | |
| --- | --- | --- | --- | --- | --- | --- | --- | --- | --- | --- | --- | --- | --- | --- | --- | --- | --- | --- |
|  | **Age** | **p-value** | **β** | **95% CI** | | |  | **p-value** | **β** | **95% CI** | | |  | **p-value** | **β** | **95% CI** | | |
| **Insulin** | |  |  |  |  |  |  |  |  |  |  |  |  |  |  |  |  |  |
|  | Childhood | **0.030** | **0.119** | **0.011** | **-** | **0.226** |  | 0.197 | 0.096 | -0.050 | - | 0.241 |  | 0.072 | 0.156 | -0.014 | - | 0.326 |
|  | Childhood and adolescence | **0.020** | **0.127** | **0.020** | **-** | **0.235** |  | 0.170 | 0.101 | -0.043 | - | 0.246 |  | **0.043** | **0.175** | **0.006** | - | **0.344** |
|  | Adolescence | **0.021** | **0.126** | **0.019** | **-** | **0.233** |  | 0.167 | 0.102 | -0.043 | - | 0.247 |  | **0.046** | **0.173** | **0.003** | - | **0.342** |
|  | Young adulthood | **0.024** | **0.129** | **0.017** | **-** | **0.241** |  | **0.047** | **0.154** | **0.002** | **-** | **0.305** |  | 0.219 | 0.110 | -0.065 | - | 0.286 |
|  | Early life | **0.016** | **0.133** | **0.024** | **-** | **0.241** |  | 0.112 | 0.119 | -0.028 | - | 0.265 |  | 0.062 | 0.163 | -0.008 | - | 0.334 |
|  |  |  |  |  |  |  |  |  |  |  |  |  |  |  |  |  |  |  |
| **Glucose** | |  |  |  |  |  |  |  |  |  |  |  |  |  |  |  |  |  |
|  | Childhood | 0.323 | -0.057 | -0.169 | - | 0.056 |  | 0.517 | -0.050 | -0.201 | - | 0.101 |  | 0.407 | -0.073 | -0.245 | - | 0.099 |
|  | Childhood and adolescence | 0.068 | -0.103 | -0.212 | - | 0.007 |  | 0.231 | -0.094 | -0.247 | - | 0.060 |  | 0.134 | -0.131 | -0.303 | - | 0.040 |
|  | Adolescence | **0.030** | **-0.120** | **-0.229** | **-** | **-0.012** |  | 0.167 | -0.109 | -0.264 | - | 0.045 |  | 0.067 | -0.161 | -0.333 | - | 0.011 |
|  | Young adulthood | **0.007** | **-0.143** | **-0.245** | **-** | **-0.040** |  | 0.155 | -0.114 | -0.271 | - | 0.043 |  | **0.009** | **-0.236** | **-0.413** | - | **-0.059** |
|  | Early life | **0.023** | **-0.125** | **-0.231** | **-** | **-0.018** |  | 0.194 | -0.102 | -0.257 | - | 0.052 |  | **0.037** | **-0.185** | **-0.357** | - | **-0.012** |
|  |  |  |  |  |  |  |  |  |  |  |  |  |  |  |  |  |  |  |
| **HDL cholesterol** | |  |  |  |  |  |  |  |  |  |  |  |  |  |  |  |  |  |
|  | Childhood | 0.235 | 0.067 | -0.044 | - | 0.178 |  | 0.813 | -0.018 | -0.166 | - | 0.130 |  | **0.048** | **0.171** | **0.002** | - | **0.341** |
|  | Childhood and adolescence | 0.244 | 0.066 | -0.045 | - | 0.178 |  | 0.791 | -0.020 | -0.168 | - | 0.128 |  | **0.048** | **0.171** | **0.002** | - | **0.341** |
|  | Adolescence | 0.248 | 0.064 | -0.045 | - | 0.174 |  | 0.747 | -0.024 | -0.172 | - | 0.124 |  | **0.049** | **0.170** | **0.001** | - | **0.340** |
|  | Young adulthood | 0.536 | 0.031 | -0.068 | - | 0.131 |  | 0.417 | -0.062 | -0.211 | - | 0.087 |  | 0.081 | 0.152 | -0.019 | - | 0.323 |
|  | Early life | 0.282 | 0.061 | -0.050 | - | 0.171 |  | 0.671 | -0.032 | -0.180 | - | 0.116 |  | **0.049** | **0.170** | **0.001** | - | **0.340** |
|  |  |  |  |  |  |  |  |  |  |  |  |  |  |  |  |  |  |  |
| **Non-HDL cholesterol** | |  |  |  |  |  |  |  |  |  |  |  |  |  |  |  |  |  |
|  | Childhood | 0.090 | 0.094 | -0.015 | - | 0.202 |  | 0.915 | 0.008 | -0.137 | - | 0.153 |  | **0.022** | **0.196** | **0.029** | - | **0.364** |
|  | Childhood and adolescence | 0.123 | 0.086 | -0.023 | - | 0.194 |  | 0.997 | 0.000 | -0.147 | - | 0.146 |  | **0.027** | **0.190** | **0.022** | - | **0.357** |
|  | Adolescence | 0.193 | 0.073 | -0.037 | - | 0.182 |  | 0.856 | -0.014 | -0.162 | - | 0.134 |  | **0.043** | **0.174** | **0.006** | - | **0.342** |
|  | Young adulthood | 0.386 | 0.049 | -0.062 | - | 0.160 |  | 0.810 | -0.018 | -0.168 | - | 0.131 |  | 0.123 | 0.133 | -0.036 | - | 0.301 |
|  | Early life | 0.174 | 0.076 | -0.033 | - | 0.185 |  | 0.897 | -0.010 | -0.157 | - | 0.138 |  | **0.039** | **0.177** | **0.009** | - | **0.344** |
|  |  |  |  |  |  |  |  |  |  |  |  |  |  |  |  |  |  |  |
| **Adiposity** | |  |  |  |  |  |  |  |  |  |  |  |  |  |  |  |  |  |
|  | Childhood | 0.150 | 0.070 | -0.025 | - | 0.165 |  | 0.146 | 0.105 | -0.037 | - | 0.247 |  | 0.395 | 0.071 | -0.092 | - | 0.234 |
|  | Childhood and adolescence | 0.234 | 0.058 | -0.038 | - | 0.154 |  | 0.185 | 0.096 | -0.046 | - | 0.238 |  | 0.541 | 0.051 | -0.112 | - | 0.215 |
|  | Adolescence | 0.340 | 0.047 | -0.050 | - | 0.145 |  | 0.238 | 0.086 | -0.056 | - | 0.227 |  | 0.689 | 0.034 | -0.131 | - | 0.198 |
|  | Young adulthood | 0.709 | 0.020 | -0.086 | - | 0.127 |  | 0.224 | 0.089 | -0.054 | - | 0.232 |  | 0.383 | -0.076 | -0.245 | - | 0.094 |
|  | Early life | 0.372 | 0.046 | -0.054 | - | 0.146 |  | 0.194 | 0.094 | -0.048 | - | 0.236 |  | 0.919 | 0.009 | -0.157 | - | 0.175 |

Additional file 1: Table S11. Associations between *nc886* epiallele methylation status (non- vs hemi-methylated) and AUC variables for different periods of early life (childhood = 6–12 years, childhood and adolescence = 6–18 years, adolescence = 12–18 years, young adulthood = 18–24 years, and early life = 6–24 years). p-values < 0.05 are indicated in boldface. Associations were analyzed one by one with a linear regression model, with sex as a covariate.

Additional file 1: Table S12. Prevalence of different *nc886* methylation status groups in individuals with type 1 diabetes in the Young Finns Study (YFS) and the Cooperative Health Research in the Region of Augsburg (KORA) cohort (n [%]).

| **Cohort/ follow-up** | | **Non-methylated** | **Intermediately methylated** | **Hemi-methylated** | **All** |
| --- | --- | --- | --- | --- | --- |
| YFS | |  |  |  |  |
|  | 2001 | 3 (37.5) | 1 (12.5) | 4 (50.0) | 8 (100.0) |
|  | 2007 | 4 (44.4) | 1 (11.1) | 4 (44.4) | 9 (100.0) |
|  | 2011 | 2 (22.2) | 2 (22.2) | 5 (55.6) | 9 (100.0) |
| KORA | |  |  |  |  |
|  | ff4 | 2 (40.0) | 1 (20.0) | 2 (40.0) | 5 (100.0) |

Additional file 1: Table S13. Associations between glycemic status groups (impaired fasting glucose (IGF) vs normoglycemia) or glucose levels and nc886 short RNA levels. The association of blood and serum nc886-3p and -5p levels (2011) with glycemic status (2011) and glucose levels (during the same follow-up as the RNA levels) was analyzed with linear regression using age, sex, smoking (yes/no), as well as leucocyte, erythrocyte, and thrombocyte count, and liver status (fatty liver yes/no) as covariates. For the 2001 and 1986 data, the analysis was performed with age, sex, and smoking (yes/no at the year of follow-up) as covariates in the model. p-values < 0.05 are indicated in boldface.

|  |  | **All** | | | | | |  | **Women** | | | | | |  | **Men** | | | | | |
| --- | --- | --- | --- | --- | --- | --- | --- | --- | --- | --- | --- | --- | --- | --- | --- | --- | --- | --- | --- | --- | --- |
|  |  | **n** | **p** | **β** | **95% CI** | | |  | **n** | **p** | **β** | **95% CI** | | |  | **n** | **p** | **β** | **95% CI** | | |
| **IFG vs normoglycemic** | | | | | |  |  |  |  |  |  |  |  |  |  |  |  |  |  |  |  |
| 2011 Blood | |  |  |  |  |  |  |  |  |  |  |  |  |  |  |  |  |  |  |  |  |
|  | nc886-3p | 781 | **0.045** | 0.162 | 0.004 | – | 0.319 |  | 424 | **0.031** | 0.262 | 0.024 | – | 0.499 |  | 357 | 0.460 | 0.081 | -0.134 | – | 0.297 |
|  | nc886-5p | 798 | 0.863 | 0.014 | -0.142 | – | 0.170 |  | 435 | 0.716 | 0.043 | -0.189 | – | 0.275 |  | 364 | 0.872 | -0.018 | -0.236 | – | 0.200 |
| 2011 Serum | |  |  |  |  |  |  |  |  |  |  |  |  |  |  |  |  |  |  |  |  |
|  | nc886-3p | 61 | **1.12E-04** | 1.047 | 0.557 | – | 1.537 |  | 31 | 0.308 | 0.482 | -0.425 | – | 1.388 |  | 30 | **1.97E-04** | 1.351 | 0.763 | – | 1.940 |
|  | nc886-5p | 120 | **0.010** | 0.544 | 0.139 | – | 0.949 |  | 72 | 0.197 | 0.407 | -0.204 | – | 1.018 |  | 48 | 0.056 | 0.601 | 0.002 | – | 1.200 |
|  |  |  |  |  |  |  |  |  |  |  |  |  |  |  |  |  |  |  |  |  |  |
| **Serum glucose levels** | | | |  |  |  |  |  |  |  |  |  |  |  |  |  |  |  |  |  |  |
| 2011 Blood | |  |  |  |  |  |  |  |  |  |  |  |  |  |  |  |  |  |  |  |  |
|  | nc886-3p | 809 | 0.268 | 0.036 | -0.027 | – | 0.099 |  | 440 | 0.411 | 0.038 | -0.052 | – | 0.128 |  | 369 | 0.563 | 0.029 | -0.069 | – | 0.127 |
|  | nc886-5p | 829 | 0.567 | -0.018 | -0.081 | – | 0.045 |  | 451 | 0.629 | -0.022 | -0.111 | – | 0.067 |  | 377 | 0.670 | -0.021 | -0.119 | – | 0.076 |
| 2011 Serum | |  |  |  |  |  |  |  |  |  |  |  |  |  |  |  |  |  |  |  |  |
|  | nc886-3p | 63 | **0.007** | 0.343 | 0.104 | – | 0.582 |  | 31 | 0.232 | 0.222 | -0.133 | – | 0.578 |  | 32 | **0.014** | 0.513 | 0.134 | – | 0.891 |
|  | nc886-5p | 125 | 0.390 | 0.074 | -0.094 | – | 0.241 |  | 75 | 0.862 | 0.020 | -0.202 | – | 0.242 |  | 50 | 0.725 | 0.057 | -0.258 | – | 0.372 |
| 2001 Serum | |  |  |  |  |  |  |  |  |  |  |  |  |  |  |  |  |  |  |  |  |
|  | nc886-3p | 40 | 0.338 | 0.127 | -0.129 | – | 0.384 |  | 21 | 0.345 | 0.166 | -0.169 | – | 0.501 |  | 19 | 0.625 | 0.116 | -0.340 | – | 0.573 |
|  | nc886-5p | 102 | 0.844 | 0.019 | -0.169 | – | 0.207 |  | 59 | 0.624 | 0.067 | -0.200 | – | 0.334 |  | 43 | 0.798 | 0.036 | -0.238 | – | 0.309 |
| 1986 Serum | |  |  |  |  |  |  |  |  |  |  |  |  |  |  |  |  |  |  |  |  |
|  | nc886-3p | 17 | **0.022** | 0.455 | 0.117 | – | 0.793 |  | 11 | 0.088 | 0.415 | 0.005 | – | 0.825 |  | 6 | 0.114 | 1.005 | 0.277 | – | 1.733 |
|  | nc886-5p | 26 | **2.18E-04** | 0.560 | 0.314 | – | 0.806 |  | 18 | **0.003** | 0.570 | 0.262 | – | 0.879 |  | 8 | 0.121 | 0.908 | 0.003 | – | 1.814 |

## Additional file 1: materials and methods

### Cohort/sample description

#### The Young Finns Study

The YFS is a multicenter follow-up study on cardiovascular risk from childhood to adulthood in Finland. The YFS was launched in 1980, when 3,596 children and adolescents (aged 3–18 years) participated in the baseline study ^2^. Thereafter, the participants have been followed with several examinations, including comprehensive risk factor assessments with major follow-ups in 1986, 2001, 2007, and 2011. The 30-year follow-up was performed in 2011, with 2,063 adults, aged 34–49 years, participating in the study. The examinations have included physical measurements, blood tests, and questionnaires. The study has been approved by the 1^st^ ethical committee of the Hospital District of Southwest Finland on September 21^st^, 2010, and by local ethical committees (1^st^ Ethical Committee of the Hospital District of Southwest Finland, Regional Ethics Committee of the Expert Responsibility area of Tampere University Hospital, Helsinki University Hospital Ethical Committee of Medicine, Research Ethics Committee of the Northern Savo Hospital District, and Ethics Committee of the Northern Ostrobothnia Hospital District). All study participants gave an informed consent, and the study was conducted according to the principles of the Declaration of Helsinki.

DNA methylation data was successful for 312 individuals included in the 1986 follow-up and for 1,714 in the 2011 follow-up, with 309 samples overlapping. The successful blood ncRNA profiling population comprises 871 individuals from the 2011follow-up, and the serum ncRNA subpopulations consist of overlapping samples from 1986 (n = 96), 2001 (n = 146), and 2011 (n = 147). Schematics of the population utilized are described in Additional file 1: Figure S1A.

#### KORA

The KORA F4 study is a follow-up of the KORA S4 study (Kooperative Gesundheitsforschung in der Region Augsburg/the Cooperative Health Research in the Region Augsburg cohort) ^3^, a population-based health survey, which collected both clinical and genetic data from the general population in the region of Augsburg and two surrounding counties between 1999 and 2001. Of the 4,261 S4 baseline study participants, aged 25–74 years, 3,080 also participated in the F4 7-year follow-up study conducted in 2006–2008. The KORA FF4 study is the second follow-up of the KORA S4 study, conducted in 2013–2014. Of the 4,261 participants of the S4 baseline study, 2,279 also participated in the FF4 14-year follow-up study. In both follow-up surveys, participants completed a lifestyle questionnaire and underwent standardized examinations with blood samples taken, as described elsewhere ^3,4^. The investigations were carried out in accordance with the Declaration of Helsinki, including written informed consent obtained from all participants. All study methods were approved by the ethics committee of the Bavarian Chamber of Physicians, Munich. DNA methylation profiling was successful for 1,727 participants of the KORA F4 study and 1,888 participants of the KORA FF4 study, with 988 individuals overlapping. Schematics of the population utilized are described in Additional file 1: Figure S1B.

#### iPSC reprogramming and cell culture

The iPSC lines were produced from skin fibroblasts with Sendai virus vectors and maintained as published before ^5^. All experiments were performed in accordance with relevant guidelines and regulations. Patients donating skin biopsies signed an informed consent after receiving both an oral and a written description of the study. A total of six iPSC lines (UTA.11201.EURCCs, EUR.10211.EURCCs, UTA.11304.EURCCs, UTA.10802.EURCCs, UTA.08203.DMs, and UTA.08102.DMs) were used in this study, and the lines were characterized as previously described ^6^. The study was approved by the Ethical Committee of Pirkanmaa Hospital District (Ethical approval no. R12123 and R08070), and written informed consent was obtained from all fibroblast donors.

The iPSC lines were maintained at 37°C in 5% CO^2^ on mitotically inactivated murine embryonic fibroblasts (MEFs; Applied StemCell, cat. no. ASF-1223). Cells were cultured in KnockOut Dulbecco’s modified Eagle medium (KO-DMEM), supplemented with 20% KnockOut Serum Replacement, 2 mM GlutaMAX, 0.1 mM 2-mercaptoethanol (2-ME) (all from Gibco), 1% nonessential amino acids (NEAA), and 50 U/ml penicillin/streptomycin (both from LONZA). The medium was supplemented with 4 ng/ml of human basic fibroblast growth factor (bFGF; R&D System).

Before hepatic differentiation, iPSCs were cultured in feeder-free conditions for 2–3 passages on culture plates coated with Geltrex (Gibco®, 1:100 dilution) and in mTeSR-1 medium (STEMCELL technologies). iPSCs were differentiated into hepatocytes by a protocol originally described by Kajiwara et al. ^7^ that was slightly modified. iPSCs were dissociated with a 5-minute Versene (Gibco) treatment and re-suspended in RPMI1640+GlutaMAX (Gibco), supplemented with 2% B27 (Gibco), 100 ng/ml Activin A (Peprotech, Cat No. 120-14E), 50 ng/ml Wnt3 (R&D systems), 50 U/ml penicillin/streptomycin, and 10 µM Rock inhibitor. Cells were seeded with 8–10 x 10^4^ /cm^2^ density. On the following day, the Rock inhibitor was replaced with 0.5 mM NaB (Sigma, B5887), and cells were cultured in this medium until they obtained definitive endoderm-specific morphology, which usually takes 4–6 days. During the second stage of differentiation, cells were cultured in KO-DMEM with 20% KO-SR, 2 mM GlutaMAX, 0.1 mM 2-mercaptoethanol, 1% NEAA, 50 U/ml penicillin/streptomycin, and 1% DMSO (Sigma, D2650) for 5–7 days until they reached the hepatoblast stage. During the third stage, cells were cultured for an additional 7–8 days in the medium prepared with HCM Hepatocyte BulletKit (Lonza, CC-3198), supplemented with 25 ng/ml HGF (PHG0254, Life Technologies) and 20 ng/ml Oncostatin M (OSM, 295-OM, R&D systems).

The hepatocytes were characterized by analyzing the mRNA expression of liver-specific genes (AFP, ALB, APOA1, ASGR1 and SERPINA1) and transcription factor FOXA2, which is important for hepatic differentiation from iPSCs (d0) and hepatocytes (d19) (Additional file 1: Material Figure S1). The expression patterns were verified with immunohistochemical staining for a subset of the genes (AFP, ALP, ASGPR) (Additional file 1: Material Figure S2). In addition, we investigated the expression patterns of the pluripotency-related miR-302 family and miR-92b-5p ^8,9^, as well as the expression of liver-specific miR-122-3p and ‑5p (Additional file 1: Material Figure S3.).

***Additional file 1:Additional file 1: Material Figure S1.*** *Expression of liver-specific genes and hepatic differentiation–related transcription factor FOXA2 in the iPSCs (d0) and hepatocytes (d19) utilized in this study. The up-regulation of the genes in hepatocytes in comparison to iPSCs is approximately 250-fold for ASGR1 and 350-fold for FOXA2, and with the other genes, the fold changes are in the magnitude of several thousands.*

**Additional file 1: Material Figure S2.** Immunohistochemical staining of the cell lines utilized in this study at d19 (hepatocytes). The detection of AFP, ALB, and ASGPR also at protein level confirms the results of the gene expression characterization and further indicates the hepatic phenotype of the differentiated cells.


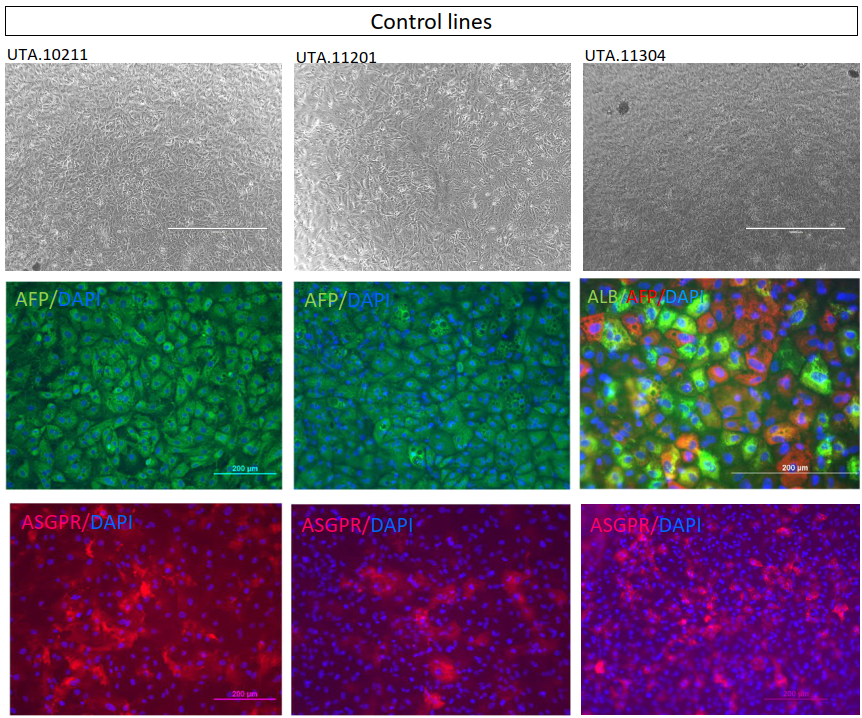


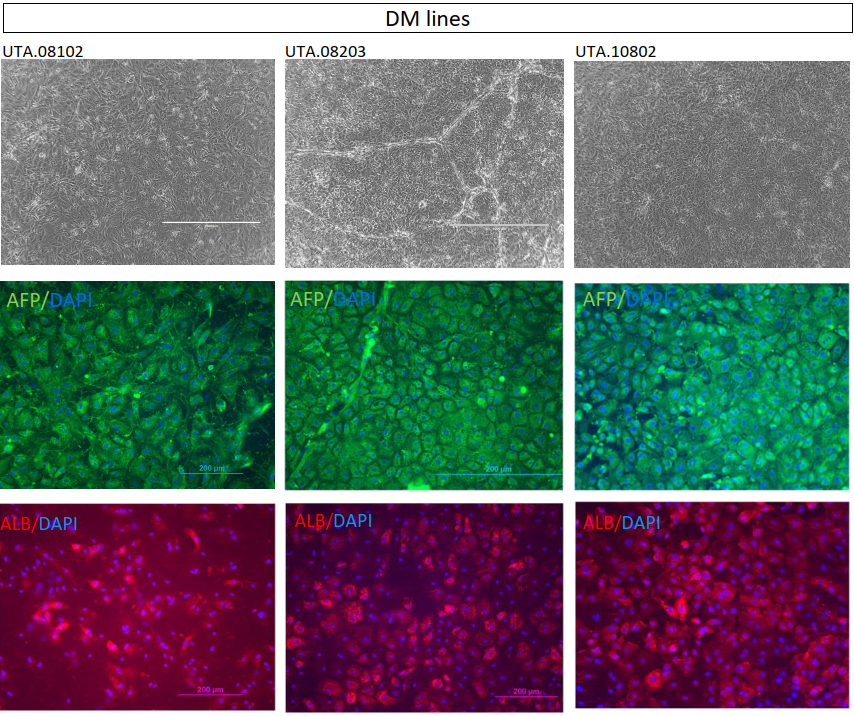


**Supplementary Material Figure S3.** Expression of pluripotency-related and liver-specific miRNAs in the cell lines utilized in this study. A systematic down-regulation of the members of pluripotency-related miR-302 members can be seen in the hepatocytes (d19) in comparison to iPSC (d0) and DE cells (d6). On average, the down-regulation is approximately -50-fold (between -169 and -10-fold). Moreover, the expression of pluripotency-related miR-92b-5p can only be detected in the iPSCs, while liver-specific miR-122-3p and ‑5p are mostly detected only in hepatocytes (d19). iPSC, induced pluripotent stem cell; DE, definitive endoderm.

The up-regulation of liver-specific genes can be seen at the mRNA (Additional file 1: Material Figure S1) and protein level (Additional file 1: Material Figure S2). In miRNAs, a systematic down-regulation of pluripotency-related miRNAs can be detected in hepatocytes. Moreover, pluripotency-related miR-92b-5p levels can only be detected in iPSCs (d0), while liver specific miR-122-3p and ‑5p levels are detected mainly in hepatocytes (miR-122-5p in one cell line already in DE cells at very low levels).

### DNA methylation profiling

#### The YFS

For the DNA methylation analysis of the YFS cohort, leukocyte DNA was obtained from EDTA blood samples collected during the 1986 and 2011 follow-ups using a Wizard® Genomic DNA Purification Kit (Promega Corporation, Madison, WI, USA) according to the manufacturer’s instructions. Genome-wide DNA methylation levels were obtained using Illumina Infinium MethylationEPIC BeadChips or Illumina Infinium HumanMethylation450 BeadChips, following the protocol by Illumina.

As regards the YFS, 181 of the 1986 samples and 188 of the 2011 samples were analyzed with Illumina Infinium HumanMethylation450 BeadChips; the 1986 samples at the Helmholtz Zentrum, Munich, Germany, and the 2011 samples in the Core Facility at the Institute of Molecular Medicine Finland (FIMM), University of Helsinki, according to the protocol by Illumina, as described earlier ^10^. Methylation signal data was preprocessed as a methylumiset object using R software (R ≥ 2.15.3) with array-specific algorithms implemented in the R package wateRmelon (40) and BMIQ (38). The resulting β values ranged linearly from 0 (non-methylated, 0%) to 1 (completely methylated, 100%). The quality of DNA samples and methylation data was carefully ensured by standard examinations with principal component analysis (PCA) and visualizations with density plots, boxplots, and dotplots.

Furthermore, 130 of the YFS 1986 samples and 1,526 of the YFS 2011 samples were analyzed with the Illumina Infinium MethylationEPIC BeadChip at Helmholtz Zentrum, Munich, Germany. Samples were applied to the arrays in a randomized order. Aliquots of 1 μg of DNA were subjected to bisulphite conversion, and a 4-μl aliquot of bisulphite-converted DNA was subjected to whole-genome amplification, followed by enzymatic fragmentation and hybridization onto an Illumina Infinium MethylationEPIC BeadChip. The arrays were scanned with the iScan reader (Illumina). All analyzed samples have a sum of detection p-values across all the probes of less than 0.01. The logged (log2) median of the methylated and unmethylated intensities of the analyzed samples clustered well visually. Furthermore, samples for which the actual sex did not match the predicted sex were excluded. Background subtraction and dye-bias normalization were performed via the noob method ^11^, followed by stratified quantile normalization. Probes with a detection p-value of more than 0.01 in 99% of the samples were filtered out. All pre-processing steps were performed using functions implemented in the *minfi* R/Bioconductor package ^12^.

#### KORA

Genome-wide DNA methylation measurement at 485,577 genomic sites was performed using the Illumina Infinium HumanMethylation450 BeadChip® (Illumina, Inc., CA) ^13^ for 1,802 **KORA F4** samples. The laboratory process has been described previously ^14^. In brief, denaturated single-stranded genomic DNA was subjected to bisulfite treatment using the EZ-96 DNA Methylation Kit (Zymo Research, Orange, CA, USA). Bisulfite-converted samples were subjected to whole-genome amplification, followed by enzymatic fragmentation and application to the BeadChips. The arrays were fluorescently stained and scanned with the Illumina HiScan SQ scanner. As readout, a methylated and an unmethylated signal count per CpG site are obtained. The counts were combined into β-values, defined as the ratio of the methylated signal intensity divided by the overall signal intensity ^13,15^: β-value = M/(M+U+α), where an offset was added as a regularization for the situation when both M and U are low, as recommended by Illumina^15^.

DNA methylation data were preprocessed following the CPACOR pipeline of Lehne et al. ^16^. Firstly, the 65 probes that represent SNPs were excluded, and background correction was performed using the R package minfi, v1.6.0 ^12^. Signals with detection p-values of ≥ 0.01, defined as the probability of a signal being detected above the background signal level as estimated from negative control probes, were removed. Similarly, signals summarized from less than three functional beads on the chip were characterized as potentially unreliable and removed from the data set. Observations with less than 95% of CpG sites providing reliable signals (75 in KORA F4) were excluded, resulting in 1,727 samples overall.

To reduce the non-biological variability between observations, data were normalized using quantile normalization on the raw signal intensities ^16^. More precisely, QN was performed on a stratification of the probe categories into 6 types, based on probe type and color channel, using the R package limma, version 3.16.5 ^17,18^. To further reduce technical variation, the principal components of the non-negative methylation control probes were calculated. The first 30 principal components were used as covariates in the regression models, as recommended by Lehne et al. ^16^*.* White blood cell proportions are important determinants of methylation in whole blood. Since cell type measurements were not available for the KORA data, proportions of selected cell types (i.e., granulocytes, monocytes, B cells, CD4+ T cells, CD8+ T cells, and natural killer cells) were estimated using the procedure proposed by Houseman et al. ^19^ and included as covariates in the models.

Genome-wide DNA methylation measurement at 865,859 genomic sites was performed using the Illumina Infinium MethylationEPIC BeadChip® (Illumina, Inc., CA, USA) for 1,928 KORA **FF4** samples. Genomic DNA (750 ng) was bisulfite-converted using the EZ-96 DNA Methylation Kit (Zymo Research, Orange, CA, USA) in two separate batches (n = 488, n = 1,440). A subsequent methylation analysis was performed on an Illumina (San Diego, CA, USA) iScan platform according to standard protocols provided by Illumina. GenomeStudio software, version 2011.1, with Methylation Module v1.9.0 was used for an initial quality control of assay performance and for the generation of methylation data export files.

Further quality control and preprocessing of the data were performed in R v3.5.1 (R Core Team (2017). R: A language and environment for statistical computing. R Foundation for Statistical Computing, Vienna, Austria. URL https://www.R-project.org/), with the package minfi v1.28.3 ^12^, and following primarily the CPACOR pipeline ^16^. Raw intensities were read into R (command read.metharray) and background-corrected (bgcorrect.illumina). Probes with detection p-values of > 0.01 were set to missing.

Before normalization, problematic samples and probes were removed. Forty samples were removed: 2 showed a mismatch between reported sex and that predicted by minfi; 33 had a median intensity of < 50% of the experiment-wide mean, or < 2000 arbitrary units; and 9 (overlap of 4 with previous) had > 5% of missing values on the autosomes. A total of 59,631 probes were removed (some overlapping multiple categories): cross-reactive probes as given in published lists (n = 44,493) ^20,21^; probes with SNPs with a minor allele frequency of > 5% at the CG position (n = 11,370) or the single base extension (n = 5,597) as given by minfi; and 5,786 with > 5% missing values. A total of 806,228 probes remained for analysis.

Quantile normalization (QN) was then performed separately on the signal intensities divided into the 6 probe types: type II red, type II green, type I green unmethylated, type I green methylated, type I red unmethylated, and type I red methylated ^16^. For the autosomes, QN was performed for all samples together; for the X and Y chromosomes, men and women were processed separately. The transformed intensities were then used to generate methylation beta values, a measure from 0 to 1 indicating the percentage of cells methylated at a given locus.

#### iPSC and methylation status verification on 12 YFS samples

Agena’s Epityper method and the MassArray Analyzer Compact Maldi-TOF-instrument were used to analyze the methylation pattern in the nc886 DMR from the 6 iPSC lines at D0 (iPSCs) and D19 (hepatocytes). This methylation analysis was performed by the Institute for Molecular Medicine Finland FIMM Technology Centre, University of Helsinki. From the iPSC lines, DNA was extracted with the NucleoSpin Tissue XS kit (Macherey Nagel) according to the manufacturer’s instructions and eluted in 20 ul of Buffer BE. DNA concentration and purity were measured with NanoDrop. In addition, 12 samples from the YFS cohort were sent for further characterization of the methylation pattern in the *nc886* epiallele. Samples were chosen to present the non-methylated (n = 3), hemi-methylated (n = 3), and intermediately methylated epiallele (n = 6) in the methylation array results for both 2011 and 1986 samples. The same DNA aliquots as those used for the DNA methylation array from the 2011 follow-up were utilized.

An overview of the method utilized can be examined at, for example, https://agenabio.com/wp-content/uploads/2015/06/51-20055R1.0-EpiTYPER-Brochure_WEB.pdf. The reactions were designed using EpiDesigner software and the [www.epidesigner.com](http://www.epidesigner.com) website. Only C cleavage was used in the reactions. Four partly overlapping amplicons that best covered the target sequence were selected (Suppmenetary Material Table S1). The sequence between positions 135415493 and 135416813 in chromosome 5 (build GRCh37) was used as the target. 500 ng of purified DNA was used as a starting material. The DNA was converted using Zymo’s EZ DNA methylation kit (cat no D5001, tube format) according to the manufacturer’s instructions. The converted product was eluted to 20 ul instead of 10 ul. After conversion, the samples were processed using Agena’s PCR Accessory and Enzyme Set (cat no 11324) and EpiTYPER Reagent, and the SpectroCHIP Set (cat no 10249) in PCR, SAP treatment and MassCLEAVE reactions. The product was de-salted with resin and spotted on SpectroCHIP arrays using the Nanodispencer-1000 instrument, followed by a Maldi-TOF run on the Agena MassArray Compact instrument. The process was performed in triplicates and as instructed by the kit provider, with the exception that the PCR step was performed twice, the second time from the PCR product due to a weak PCR product during the first PCR round. A subset of the samples was checked on Flash gels (Lonza) for quality of the PCR product before proceeding to the next step of the workflow. The primer sequences are listed below in Additional file 1: Material Table S1. HIGH and LOW methylation commercial controls (EpigenDx), a converted and un-converted lab-specific sample, and water were used as controls. The data was analyzed using Epityper 1.3 (Agena) software.

Out of the analyzed four amplicons, the results with Amplicon 4 were suitable for further processing. For each sample, and for each of the 10 CpG sites analyzed from this amplicon, the three replicates were visually inspected and clear outliers removed. Averages for the replicates for each CpG were then calculated, and the median of these averages was treated as the median methylation in the *nc886* epiallele.

***Additional file 1: Material Table S1.*** *Amplicons used in the study. Final results are based solely on the results of the Amplicon 4 overlapping cg13581155, cg11978884, cg11608150, and cg06478886 from the array data.*

| Amplicon name | Amplicon 1 |
| --- | --- |
| Length | 231 |
| Left Primer | aggaagagagTGTGTATGTTTTTGTGTATTAAGTTATTT |
| Right Primer | cagtaatacgactcactatagggagaaggctATTCCAAATTTCTTCCTCCCTAAC |
|  |  |
| Amplicon name | Amplicon 4 |
| Length | 342 |
| Left Primer | aggaagagagGTTAGGGAGGAAGAAATTTGGAAT |
| Right Primer | cagtaatacgactcactatagggagaaggctCAAAAAATATAAAAACTAACCACTAATCTT |
|  |  |
| Amplicon name | Amplicon 5 |
| Length | 460 |
| Left Primer | aggaagagagAAGATTAGTGGTTAGTTTTTATATTTTTTG |
| Right Primer | cagtaatacgactcactatagggagaaggctAATAAATAAATTTTACCCCCTTCCA |
|  |  |
| Amplicon name | Amplicon 9 |
| Length | 337 |
| Left Primer | aggaagagagGTGTTTTGGAGGGATTTTTAGTTTT |
| Right Primer | cagtaatacgactcactatagggagaaggctCCCCACCCAAAAAAAATTTACTA |

### RNA isolation and sncRNA arrays

***Blood***

RNA was isolated and miRNA and gene expression profiling was performed as described previously ^22^. In brief, whole blood was collected into PaXgene Blood RNA Tubes (PreAnalytix) and isolated with a PAXgene Blood microRNA Kit (Qiagen), including the DNase Set with the QiaCube according to the manufacturer’s instructions. MicroRNA expression profiling was performed with the TaqMan® OpenArray® MicroRNA Panel (Applied Biosystems), containing 758 microRNAs, including nc886-3p (Assay ID 002194) and nc886-3p (Assay ID 002193), using the standard protocol. MicroRNA panels were loaded using the AccuFill System and run with the QuantStudio 12K Flex (Applied Biosystems). Primary data analysis was performed with Expression Suite Software version 1.0.1. U6 snRNA, RNU44, and RNU48 were used as housekeeping small RNAs. After quality control and removal of outlier miRNA, profiling was successful on 871 samples. To correct for batch effects, principal components analysis was performed for miRNA expression data. The data was adjusted for 10 of the first 20 principal components from the principal component analysis.

***Serum***

MicroRNAs were isolated from 200 µl of serum with the miRNeasy Serum/Plasma Kit (Qiagen), including the DNase Set, using the QiaCube. MicroRNA expression profiling was performed with the same TaqMan® OpenArray® MicroRNA Panel (Life Technologies) as with whole blood, but the scaled-down Low Sample Protocol was used for serum samples. As opposed to standard protocol, 10 µl of the miRNA eluate was used in cDNA synthesis for both A and B pools of Megaplex, and 7.5 µl of cDNA was preamplified (total reaction volume 18 µl) and diluted to a final dilution ratio of 1:10 with nuclease-free water and TE buffer. Also, for serum data, only assays with an Amplification score of > 1 and Cq Confidence of > 0.7 were accepted. The serum data was normalized with a global mean normalization approach in R language. The approach involves the calculation of a normalization factor as the global mean of all expressed miRNAs per sample. Furthermore, between-sample normalization was carried out with the quantile normalization method. Batch correction, when needed, was performed with ComBat.

***iPSC***

RNA from the cell lines was extracted with a Qiagen miRNeasy mini kit, and microRNAs were profiled using the standard protocol, with the exception of running PreAmplification-PCR with 16 instead of 12 cycles. MicroRNA panels were loaded using the AccuFill System and run with the QuantStudio 12K Flex (Applied Biosystems). Only assays with an Amplification score of > 1 and Cq Confidence of > 0.7 were accepted, and data was normalized with the global mean normalization approach in R language.

### Gene expression profiling

***Genome-wide gene expression profiling from YFS 2011 blood samples***

The expression levels were analyzed with an Illumina HumanHT-12 version 4 Expression BeadChip (Illumina Inc.). Utilizing the same RNA sample for both mRNA and sncRNA expression profiling, 200 ng of RNA was reverse-transcribed into cDNA and biotin-UTP-labeled using the Illumina TotalPrep RNA Amplification Kit (Ambion); 1,500 ng of cDNA was then hybridized onto the Illumina HumanHT-12 v4 Expression BeadChip. The BeadChips were scanned with the Illumina iScan system. Raw Illumina probe data was exported from Genomestudio and analyzed in R (http://www.r-project.org/), using the Bioconductor (http://www.bioconductor.org/) packages. The expression data was processed using nonparametric background correction, followed by quantile normalization with control and expression probes, using the neqc function in the limma package and log2 transformation. The data processing is described in more detail in the article by Elovainio et al. ^23^. The expression analysis was successful in 743 of the 871 samples with a miRNA expression profile.

***nc886-102nt quantification from YFS serum and blood samples and from iPSC samples***

nc886-102nt was quantified with qRT-PCR from the serum and blood of 50 individuals from the YFS, for whom successful nc886-3p and -5p expression profiling and methylation data was available. Samples from all of the 6 iPSC lines (two technical replicates per cell line) from d0 were also included in the quantification. nc886-102nt was quantified with the Applied Biosystems assay for vtRNA2-1 (Hs04273370_s1), and B2M (Hs00187842_m1) was utilized as a reference gene. From blood and cell culture samples, 500 ng of RNA as well as 7 µl of eluate from serum samples were utilized in the RT reaction, and the reaction was performed with the High-Capacity cDNA Reverse Transcription Kit (Thermo Fisher) according to the manufacturer’s instructions. qPCR was run with QuantStudio 12K Flex (Applied Biosystems), following the protocol for TaqMan™ Fast Advanced Master Mix (Thermo Fisher). Cq-values higher than 35 were discarded, and only replicates with an Amplification score of > 1 and Cq Confidence of > 0.7 were accepted for further analysis.

### Genome-wide genotyping in the YFS

Genomic DNA was extracted from peripheral blood leukocytes using a commercially available kit and a Qiagen BioRobot M48 Workstation according to the manufacturer’s instructions (Qiagen, Hilden, Germany). Genotyping was performed using a custom-built Illumina Human 670 k BeadChip at the Welcome Trust Sanger Institute. Genotypes were called using the Illuminus clustering algorithm. Samples that failed the Sanger genotyping pipeline QC criteria (i.e., duplicated samples, heterozygosity, low call rate, or Sequenom fingerprint discrepancy) were excluded. Similarly, samples with gender discrepancy, a low genotyping call rate (< 0.95) and possible relatedness (pi-hat > 0.2) were excluded. Short Nucleotide Polymorphisms (SNPs) were filtered based on an Hardy-Weinberg equilibrium test (p ≤ 1e-06), missingness test (call rate < 0.95), and frequency test (minor allele frequency < 0.01). After quality control, 546,677 genotyped SNPs were available for further analysis. Genotype imputation was performed using Minimac3 ^24^ and the 1000G phase3 reference set on the Michigan Imputation Server. Autosomes and sex chromosomes were phased using Eagle ^25^ and SHAPEIT ^26^, respectively.

### Clinical and biochemical measurements

#### YFS

Weight and height were measured, and BMI was calculated as weight(kg)/(height(m))². Waist circumference was measured to the nearest 0.1 cm. For the biochemical measurements, venous blood samples were drawn from the right antecubital vein after an overnight fast, and serum was separated, aliquoted, and stored at -70°C until analysis. Glucose, cholesterol, and triglyceride concentrations were measured with Glucose, Cholesterol, and Triglycerides System Reagent (Beckman Coulter Biomedical). The serum triglyceride concentration was assayed using the enzymatic glycerol kinase–glycerol phosphate oxidase method (Beckman Coulter Biomedical). Serum total cholesterol levels were measured by the enzymatic cholesterol esterase–cholesterol oxidase method (Beckman Coulter Biomedical). The same reagent was used for estimating HDL cholesterol levels after the precipitation of low-density lipoprotein (LDL) and very low-density lipoprotein (VLDL) with dextran sulfate- Mg2+. LDL cholesterol was calculated indirectly using the Friedewald formula. Non-high-density lipoprotein (non-HDL) cholesterol was calculated as total cholesterol minus HDL cholesterol.

Insulin levels were measured with a microparticle enzyme immunoassay kit (Abbott Laboratories, Chicago, IL). Serum alanine aminotransferase (ALT), aspartate aminotransferase (AST), and gamma-glutamyl transferase (GT) concentrations were measured with System Reagent (Beckman Coulter Biomedical). For HbA1c fraction measurement, the concentration of total hemoglobin was determined colorimetrically, after which the concentration of HbA1c was measured immunoturbidimetrically. These two concentrations were used to calculate the HbA1c percentage (HbA1c%). Insulin levels were measured by a microparticle enzyme immunoassay kit. Subjects were categorized into the normoglycemic, impaired fasting glucose, and T2D groups, based on fasting serum glucose and HbA1c according to the WHO criteria ^27^ and a self-reported diagnosis of T2D by a physician. Subjects with type 1 diabetes were excluded from the analysis.

The repeatedly measured data from the Young Finns Study were leveraged to describe long-term trends of weight, height, BMI, adiposity, as well as serum glucose, insulin, and lipid levels, as described earlier ^28^. In brief, we utilized several risk factor measurements from 1980 to 2011 to calculate the area under the curve (AUC) separately for each measurement. First, subject-specific curves for the measurements were estimated by mixed model regression splines ^29^. Then, similar to the approach of Lai et al. ^30^, AUCs were evaluated for each measurement. Individual AUCs were then utilized to create estimate values for the measurements for each age category from childhood to adulthood. To use all available repeatedly measured exposure data for continuous variables, we estimated subject-specific curves for cardiovascular risk factors with mixed model regression splines ^29^. The area under the curve (AUC) for continuous risk variables was evaluated to indicate a long-term burden of each measured attribute ^30^. The AUC variables were defined separately for childhood (6–12 years), childhood and adolescence (6–18 years) adolescence (12–18 years), young adulthood (18–24 years), and early life (6–24 years). For adulthood, the measurements from 2001, 2007, and 2011 were also utilized.

#### KORA

KORA FF4 study participants underwent an extensive standardized medical examination, including the collection of blood samples. All measurement procedures were described in detail elsewhere ^31^.

Weight and BMI were measured at the study center in a standardized fashion by trained examiners ^31,32^. Body weight was measured in light clothing to the nearest 0.1 kg and height to the nearest 0.5 cm. Body mass index (BMI) was calculated as weight (kg)/height^2^ (m^2^).

Glucose, HDL and LDL cholesterol, as well as triglyceride levels were measured from fresh fasting serum by enzymatic, colorimetric methods using GLU, LDLC, HDLC, and TRIG Flex assays on a Dimension Vista 1500 instrument (Siemens Healthcare Diagnostics Inc., Newark, USA), or GLUC3, LDL_C, HDLC3, and TRIGL assays on a Cobas c701/702 instrument (Roche Diagnostics GmbH, Mannheim, Germany) ^33^. Serum insulin concentrations were assessed by a solid-phase enzyme-labeled chemiluminescent immunometric assay on an Immulite 2000 systems analyzer (Siemens) or by an electrochemiluminescence immunoassay on a Cobas e602 instrument (Roche) ^34^. The measurement instrument and assays changed from Siemens to Roche during the study. Calibration formulas were developed using 122 (194 for insulin) KORA FF4 samples that were measured with both methods during the time of the change ^33,34^ (PMID. The Siemens measurement results were calibrated to the Roche measurements using the following formulas [all units in mg/dl]: HDL_Cholesterol_Roche = 2.40 + HDL_Cholesterol_Siemens * 1.12; LDL_Cholesterol_Roche = antilog (-0.13328 + log LDL_Cholesterol_Siemens * 1.03051); Triglycerides_Roche = 4.97073 + Triglycerides_Siemens * 0.90732 (PMID: 28141837); Insulin_Roche = 7.842 pmol/L + Insulin_Siemens × 1.016. The homeostasis model assessment insulin resistance (HOMA-IR) was calculated as fasting insulin (in pmol/l) × fasting glucose (in mmol/l) ÷ 135 ^34^.

No calibration was needed for the glucose assessment because the double measurements were very similar so that the intercept and the slope of the Passing-Bablok regression used for calibration were estimated to be zero and one, respectively ^33^.

HbA1c was measured in hemolyzed whole blood using the cation-exchange high performance liquid chromatographic, photometric VARIANT II TURBO HbA1c Kit—2.0 assay on a VARIANT II TURBO Hemoglobin Testing System (Bio-Rad Laboratories Inc., Hercules, CA, USA) ^33^.

All participants without known diabetes were assigned to undergo a standard 75 g oral glucose tolerance test (OGTT). Blood samples were taken without stasis after an overnight fast of at least 8 h and 2 h after glucose solution intake; further details have been described elsewhere ^33^. Serum fasting glucose (FG) and 2-h postload glucose (2HG) levels were measured using an enzymatic colorimetric method (Dimension Vista 1500, Siemens Healthcare Diagnostics, Eschborn, Germany or Cobas c702, Roche Diagnostics GmbH, Mannheim, Germany), and fasting insulin (FI) and 2-h postload insulin (2HI) values were measured by a solid-phase enzyme-labeled chemiluminescent immunometric assay (Immulite 2000 Xpi, Siemens Healthcare Diagnostics, Eschborn, Germany) or by an electrochemiluminescence immunoassay (Cobas e 602, Roche Diagnostics GmbH, Mannheim, Germany).

Individuals with an OGTT value of ≥ 7.0 mmol/l fasting or ≥ 11.1 mmol/l 2-h glucose were classified with diabetes mellitus. Prediabetes was defined as having impaired fasting glucose (5.6–6.9 mmol/l fasting glucose), impaired glucose tolerance (7.8–11.0 mmol/l 2-h glucose) or the combination of both. Normal glucose tolerance (NGT) was considered a fasting glucose value of < 5.6 mmol/l and a 2-h glucose OGTT value of < 7.8 mmol/l. Previously known type 2 diabetes was defined as a self-report that could be validated by the responsible physician or a medical records review, or as current use of glucose-lowering medication.

The liver enzymes GT, AST, and ALT were analyzed according to the recommendations of the International Federation of Clinical Chemistry (IFCC) from 1983, including the optimization of substrate concentrations, the employment of NaOH, glycylglydine buffer, and sample start. Pyridoxal phosphate was applied in the assessment of ALT and AST ^35^.

### Birth family’s socioeconomic factors and maternal age at birth in the YFS

Maternal age at childbirth was calculated by subtracting the age of the YFS participant in 1980 from the mother’s reported age at the same follow-up. Participants were divided into groups according to maternal age at childbirth: ≤ 20, 21–35, and ≥ 36 years. The occupation of the parents was obtained by a questionnaire in 1980 and classified as (I) upper non-manual, (II) lower non-manual, (III) upper manual, (IV) lower manual, and (F) farmers ^36^. Parental education in 1980 was determined on the basis of school years completed, and it was classified into three groups: (I) less than 9 years, (II) 9–12 years, and (III) more than 12 years. The information on the parent with the most years of schooling was used in the study ^37^. Family income was divided into three groups: (I) lowest quartile, (II) interquartile range, and (III) the highest quartile.

### Statistical analysis

#### All statistical analyses were performed with R statistical software (v.3.5.1) or with IBM SPSS statistics 25, if not otherwise stated.

#### EWAS

An EWAS on whole blood nc886-3p and -5p levels separately was performed with the YSF samples profiled with an EPIC array (n = 1,526). A multiple linear regression analysis with nc886-3p as outcome and methylation beta values as predictor—adjusted with age, sex, white blood cell proportions estimated with the Houseman method ^19^, and the first 30 principal components of the control probes to control for technical covariates was performed. The exact same analysis was also performed on nc886-5p. Statistical significance was set at p < 5*10^-8.^

#### Clustering of samples based on nc886 methylation

Clustering was performed separately for samples profiled with Illumina 450K and EPIC arrays, and separately for the follow-up studies (1986 and 2011 in the YFS and F4 and FF4 in the KORA). The clustering was based on 14 CpG sites, located within 1000 bp around the coding region of nc886, that show a bimodal distribution of methylation values (cg07158503, cg04515200, cg13581155, cg11978884, cg11608150, cg06478886, cg04481923, cg18678645, cg06536614, cg25340688, cg26896946, cg00124993, cg08745965, and cg18797653; Additional file 1: Figure S2). Samples were clustered using hierarchical clustering (R, hclust with default settings). Clustering was visualized as a dendogram, based on which the dataset was divided. For each group, the median methylation value of this region was used to decide which groups were non-, intermediately, or hemi-methylated. Median values around or below 0.20 were interpreted as representing non-methylated individuals, and median values around or above 0.45 were interpreted as hemi-methylated, with the remaining groups interpreted as intermediately methylated. For each group, the correct classification was verified by histograms of the methylation values of the CpG sites used in the clustering.

Similarly, the methylation values of the 14 CpG sites were retrieved from the data of Rhead et al.^1^ (GSE131989), and median methylation values were calculated and samples clustered for each cell type separately for the 59 individuals who could be reliably mapped as individual participants according to their phenodata.

#### Stability of the methylation status

The stability of the methylation status was evaluated with the 309 individuals from the YFS and the 988 individuals with KORA with repeated DNA methylation profiling available. Median methylation values for the 14 CpG sites selected for the clustering of individuals were calculated, and differences between the medians of individuals were calculated. In addition, the number of individuals changing groups between the time points was investigated. Two individuals had a discrepancy in the *nc886* epiallele methylation status. These samples had low methylation levels (β < 0.1) at one time point and high methylation levels (β ~ 0.5) at the other. As there were only two samples in each cohort, and as we were unable to determine whether this is a true biological phenomenon or a sampling or handling error, these samples were discarded from further analyses.

#### Blood and serum nc886-3p and -5p levels of individuals in different nc886 methylation groups

The nc886 RNA expression data was analyzed with the ΔΔCq method. Fold changes for nc886-3p, -5p, and ‑102nt were calculated separately for the ncRNAs in the YFS blood samples and different serum subpopulations, using the median of the hemi-methylated group as the reference. For iPSCs, the median of all samples at d0 was used as the reference value. Between-group differences were evaluated with the Mann-Whitney U test and trend over status groups with the Kruskal-Wallis test. Statistical significance was set at p = 0.05.

#### GWASs on nc886 methylation and miRNA expression levels

All association analyses were performed using regression models in SNPTEST version 2.5.4. Genome-wide association analyses on methylation status (non-methylated vs hemi-methylated) were performed with a logistic regression model adjusted for age, sex, smoking status, estimated blood cell proportions, and the first 5 principal components of the methylation analysis. GWAS results on the 18 CpG sites located in the *nc886* DMR were retrieved from the Genetics of DNA Methylation Consortium (GoDMC) database (<http://mqtldb.godmc.org.uk/index.php>)^39^, presenting GWAS results for each CpG from over 30,000 individuals. As all of the discovered variations were cis and located within ± 1Mb of the *nc886* locus, we performed an association analysis on the YFS data within these genomic boundaries for the *nc886* DMR CpGs. Only variations with a minor allele frequency of 1% were investigated in the YFS data

Genome-wide association analyses for miR886-3p and miR886-5p each were performed with linear regression models adjusted for age, sex, smoking status (yes/no), methylation status (non-methylated/intermediately methylated/hemi-methylated), and fasting status (yes/no). Statistical significance for all of the above-mentioned association analyses was set at p < 5*10^-8.^

#### Determinates of nc886 epiallele methylation status

The associations of parental income, education, and occupational status, as well as the maternal age groups with the prevalence of hemi-methylated and non-methylated individuals were assessed by comparing groups one by one with the Chi squared test. Statistical significance was set at p = 0.05.

#### Differential gene expression and gene set analysis (pathway analysis) for nc886 methylation status

A differential gene expression analysis of genome-wide gene expression data with respect to nc886 methylation status (non- vs hemi-methylated) was performed using the limma and Biobase R/Bioconductor packages (18, 39). The analysis was adjusted with age, sex, smoking status, fasting status, blood cell proportions (leukocytes, erythrocytes and trombocytes), the first 2 principal components, and gene expression array–based technical covariates (plate, well and position). A gene set analysis of the gene expression data with respect to the two nc886 methylation categories was carried out with curated gene sets downloaded from Molecular Signature Database (MSigDB) on April 8, 2020, using the threshold-free gene set analysis method mGSZ ^40^. Statistical significance was set at FDR < 0.05.

#### Target mRNA–driven GSEA for nc886-3p and -5p target genes

The predicted mRNA targets of nc886-3p and nc886-5p were included in the correlation analysis if they were recognized as targets for hsa-miR-886-3p or ‑5p by microRNA.org. Correlations between nc886-3p and ‑5 and their predicted targets were calculated separately with Spearman’s correlation, and individual correlations with an FDR of < 0.05 were considered significant. Predicted target mRNAs that correlated at the level of p < 0.05 with the levels nc886-3p or ‑5p were selected for further pathway enrichment analysis. The overlaps between the selected target mRNAs and gene set in KEGG and BIOCARTA were analyzed in a molecular signature database. Gene sets containing at least 5 of the selected target mRNAs and with an FDR of < 0.05 were considered to be enriched with the targets that correlated with the expression of the ncRNA. The analysis was first run separately for genes correlating with nc886-3p and ‑5p and then together with all target mRNAs correlating with at least one of the ncRNAs.

#### Association between clinical and biochemical measurements and nc886 epiallele methylation status

All regression analyses were performed with R language, and all continuous variables were inverse-normal-transformed. Associations between the *nc886* epiallele methylation status (non- vs hemi-methylated) and the YFS yearly estimates of height, weight, BMI, adiposity, HDL, LDL, total cholesterol, non-HDL cholesterol, triglycerides, glucose, insulin, ALT, AST, and GT were assessed one by one with a linear regression model with sex as an covariate. The analysis was also repeated separately for men and women without covariates. A linear regression model with age and sex as covariates was used to analyze the association of *nc886* epiallele methylation status and actual measures of height, weight, BMI, HDL, LDL, total cholesterol, non-HDL cholesterol, triglycerides, glucose, and insulin from the YFS 2001, 2007, and 2011 follow-ups and KORA FF4, and sex-stratified analyses were performed with age as the only covariate in the model. Statistical significance was set at p < 0.05.

#### Association of clinical and biochemical measurements with nc886-3p and ‑5p levels

Associations between blood and serum nc886-3p and -5p levels (2011 samples) and the metabolic traits (one by one: height, weight, BMI, adiposity, HDL, LDL, total cholesterol, non-HDL cholesterol, triglycerides, glucose, insulin, ALT, AST, and GT), were analyzed with linear regression, using age, sex, smoking (yes/no), as well as leucocyte, erythrocyte, and thrombocyte count, and liver status (fatty liver yes/no) as covariates. For serum nc886-3p and -5p, a similar regression analysis was performed for the 1986 and 2001 measurements separately, predicting the metabolic traits (one by one: height, weight, BMI, adiposity, HDL, LDL, total cholesterol, non-HDL cholesterol, triglycerides, glucose, insulin, ALT, AST, and GT) measured during the same follow-up (with the exception of liver enzymes, which were not measured in 1986), with age, sex, and smoking (yes/no at the year of follow-up) as covariates in the model. The association between impaired fasting glucose and nc886-3p and -5p levels in 2011 was analyzed with a regression model (impaired fasting glucose yes/no 2011, individuals with T2D or T1D were discarded from the analysis) using age, sex, smoking, blood cell proportions, and liver status as covariates. Statistical significance was set at p < 0.05.

## Additional file 1: references

1. B. R, C. H, M. C, et al. DNA methylation profiles that distinguish rheumatoid arthritis from osteoarthritis in fibroblast-like synoviocytes can be detected in immune cells from peripheral blood. *Arthritis and Rheumatology*. Published online 2014. doi:http://dx.doi.org/10.1002/art.38914

2. Raitakari OT, Juonala M, Ronnemaa T, et al. Cohort profile: the cardiovascular risk in Young Finns Study. *Int J Epidemiol*. 2008;37(6):1220-1226.

3. Holle R, Happich M, Lowel H, Wichmann HE, Group MS. KORA--a research platform for population based health research. *Gesundheitswesen (Bundesverband der Arzte des Offentlichen Gesundheitsdienstes (Germany))*. 2005;67 Suppl 1:S19-25. doi:10.1055/s-2005-858235 [doi]

4. Wichmann HE, Gieger C, Illig T. KORA-gen - Resource for population genetics, controls and a broad spectrum of disease phenotypes. *Gesundheitswesen*. Published online 2005. doi:10.1055/s-2005-858226

5. Manzini S, Viiri LE, Marttila S, Aalto-Setälä K. A Comparative View on Easy to Deploy non-Integrating Methods for Patient-Specific iPSC Production. *Stem cell reviews*. 2015;11(6):900-908. doi:10.1007/s12015-015-9619-3

6. Kiamehr M, Viiri LE, Vihervaara T, et al. Lipidomic profiling of patient-specific iPSC-derived hepatocyte-like cells. *Disease Models & Mechanisms*. 2017;10(9).

7. Kajiwara M, Aoi T, Okita K, et al. Donor-dependent variations in hepatic differentiation from human-induced pluripotent stem cells. *Proceedings of the National Academy of Sciences of the United States of America*. 2012;109(31):12538-12543. doi:10.1073/pnas.1209979109

8. Lakshmipathy U, Davila J, Hart RP. MiRNA in pluripotent stem cells. *Regenerative Medicine*. Published online 2010. doi:10.2217/rme.10.34

9. Kumar S, Espinosa EC, Leandro AC, Curran JE, Blangero J. microRNA and mRNA interactions in induced pluripotent stem cell reprogramming of lymphoblastoid cell lines. *American journal of stem cells*. Published online 2019.

10. Kananen L, Marttila S, Nevalainen T, et al. The trajectory of the blood DNA methylome ageing rate is largely set before adulthood: evidence from two longitudinal studies. *Age (Dordrecht, Netherlands)*. 2016;38(3):65-016-9927-9. Epub 2016 Jun 14. doi:10.1007/s11357-016-9927-9 [doi]

11. Triche TJ, Weisenberger DJ, Van Den Berg D, Laird PW, Siegmund KD. Low-level processing of Illumina Infinium DNA Methylation BeadArrays. *Nucleic Acids Research*. Published online 2013. doi:10.1093/nar/gkt090

12. Aryee MJ, Jaffe AE, Corrada-Bravo H, et al. Minfi: A flexible and comprehensive Bioconductor package for the analysis of Infinium DNA methylation microarrays. *Bioinformatics*. Published online 2014. doi:10.1093/bioinformatics/btu049

13. Bibikova M, Barnes B, Tsan C, et al. High density DNA methylation array with single CpG site resolution. *Genomics*. Published online 2011. doi:10.1016/j.ygeno.2011.07.007

14. Zeilinger S, Kuhnel B, Klopp N, et al. Tobacco smoking leads to extensive genome-wide changes in DNA methylation. *PloS one*. 2013;8(5):e63812. doi:10.1371/journal.pone.0063812 [doi]

15. Du P, Zhang X, Huang CC, et al. Comparison of Beta-value and M-value methods for quantifying methylation levels by microarray analysis. *BMC Bioinformatics*. Published online 2010. doi:10.1186/1471-2105-11-587

16. Lehne B, Drong AW, Loh M, et al. A coherent approach for analysis of the Illumina HumanMethylation450 BeadChip improves data quality and performance in epigenome-wide association studies. *Genome Biology*. Published online 2015. doi:10.1186/s13059-015-0600-x

17. Smyth GK, Michaud J, Scott HS. Use of within-array replicate spots for assessing differential expression in microarray experiments. *Bioinformatics*. Published online 2005. doi:10.1093/bioinformatics/bti270

18. Ritchie ME, Phipson B, Wu D, et al. Limma powers differential expression analyses for RNA-sequencing and microarray studies. *Nucleic Acids Research*. Published online 2015. doi:10.1093/nar/gkv007

19. Houseman EA, Accomando WP, Koestler DC, et al. DNA methylation arrays as surrogate measures of cell mixture distribution. *BMC bioinformatics*. 2012;13:86. doi:10.1186/1471-2105-13-86 [doi]

20. Pidsley R, Zotenko E, Peters TJ, et al. Critical evaluation of the Illumina MethylationEPIC BeadChip microarray for whole-genome DNA methylation profiling. *Genome Biology*. Published online 2016. doi:10.1186/s13059-016-1066-1

21. McCartney DL, Walker RM, Morris SW, McIntosh AM, Porteous DJ, Evans KL. Identification of polymorphic and off-target probe binding sites on the Illumina Infinium MethylationEPIC BeadChip. *Genomics Data*. Published online 2016. doi:10.1016/j.gdata.2016.05.012

22. Raitoharju E, Seppala I, Lyytikainen LP, et al. Blood hsa-miR-122-5p and hsa-miR-885-5p levels associate with fatty liver and related lipoprotein metabolism-The Young Finns Study. *Scientific reports*. 2016;6:38262. doi:10.1038/srep38262 [doi]

23. Elovainio M, Taipale T, Seppala I, et al. Activated immune-inflammatory pathways are associated with long-standing depressive symptoms: Evidence from gene-set enrichment analyses in the Young Finns Study. *Journal of psychiatric research*. 2015;71:120-125. doi:10.1016/j.jpsychires.2015.09.017 [doi]

24. Fuchsberger C, Abecasis GR, Hinds DA. Minimac2: Faster genotype imputation. *Bioinformatics*. Published online 2015. doi:10.1093/bioinformatics/btu704

25. Loh PR, Palamara PF, Price AL. Fast and accurate long-range phasing in a UK Biobank cohort. *Nature Genetics*. Published online 2016. doi:10.1038/ng.3571

26. Delaneau O, Zagury JF, Marchini J. Improved whole-chromosome phasing for disease and population genetic studies. *Nature Methods*. Published online 2013. doi:10.1038/nmeth.2307

27. Organization WH. *Global Report on Diabetes*.; 2016.

28. Koskinen JS, Kytö V, Juonala M, et al. Childhood risk factors and carotid atherosclerotic plaque in adulthood: The Cardiovascular Risk in Young Finns Study. *Atherosclerosis*. Published online 2020. doi:10.1016/j.atherosclerosis.2019.11.029

29. Welham SJ. Smoothing spline models for longitudinal data. In: *Longitudinal Data Analysis*. ; 2008. doi:10.1201/9781420011579.ch11

30. Lai CC, Sun D, Cen R, et al. Impact of long-term burden of excessive adiposity and elevated blood pressure from childhood on adulthood left ventricular remodeling patterns: The bogalusa heart study. *Journal of the American College of Cardiology*. Published online 2014. doi:10.1016/j.jacc.2014.05.072

31. Rathmann W, Haastert B, Icks A, et al. High prevalence of undiagnosed diabetes mellitus in Southern Germany: Target populations for efficient screening. The KORA survey 2000. *Diabetologia*. Published online 2003. doi:10.1007/s00125-002-1025-0

32. Rathmann W, Haastert B, Icks A, et al. Sex differences in the associations of socioeconomic status with undiagnosed diabetes mellitus and impaired glucose tolerance in the elderly population: The KORA Survey 2000. *European Journal of Public Health*. Published online 2005. doi:10.1093/eurpub/cki037

33. Kowall B, Rathmann W, Stang A, et al. Perceived risk of diabetes seriously underestimates actual diabetes risk: The KORA FF4 study. *PLoS ONE*. Published online 2017. doi:10.1371/journal.pone.0171152

34. Huth C, von Toerne C, Schederecker F, et al. Protein markers and risk of type 2 diabetes and prediabetes: a targeted proteomics approach in the KORA F4/FF4 study. *European Journal of Epidemiology*. Published online 2019. doi:10.1007/s10654-018-0475-8

35. Rückert IM, Heier M, Rathmann W, Baumeister SE, Döring A, Meisinger C. Association between markers of fatty liver disease and impaired glucose regulation in men and women from the general population: The KORA-F4-study. *PLoS ONE*. Published online 2011. doi:10.1371/journal.pone.0022932

36. Leino M, Porkka KVK, Raitakari OT, Laitinen S, Taimela S, Viikari JSA. Influence of parental occupation on coronary heart disease risk factors in children. The Cardiovascular Risk in Young Finns Study. *International Journal of Epidemiology*. Published online 1996. doi:10.1093/ije/25.6.1189

37. Leino M, Raitakari OT, Porkka KVK, Helenius HYM, Viikari JSA. Cardiovascular risk factors of young adults in relation to parental socioeconomic status: The Cardiovascular Risk in Young Finns Study. *Annals of Medicine*. Published online 2000. doi:10.3109/07853890009011764

38. Huber W, Carey VJ, Gentleman R, et al. Orchestrating high-throughput genomic analysis with Bioconductor. *Nature Methods*. Published online 2015. doi:10.1038/nmeth.3252

39. Min JL, Hemani G, Hannon E, Dekkers KF, Castillo-Fernandez J, et al. Genomic and phenomic insights from an atlas of genetic effects on DNA methylation. medRxiv preprint. Available at : https://doi.org/10.1101/2020.09.01.20180406. Deposited September 3, 2020

40. Mishra P, Törönen P, Leino Y, Holm L. Gene set analysis: Limitations in popular existing methods and proposed improvements. *Bioinformatics*. Published online 2014. doi:10.1093/bioinformatics/btu374
